# Field Test of the World Health Organization Multi-Professional Patient Safety Curriculum Guide

**DOI:** 10.1371/journal.pone.0138510

**Published:** 2015-09-25

**Authors:** Donna Farley, Hao Zheng, Eirini Rousi, Agnès Leotsakos

**Affiliations:** 1 Service Delivery and Safety Department, World Health Organization, Geneva, Switzerland; 2 Division of Medical Humanities and Behavioral Sciences, Tongji University School of Medicine, Shanghai, China; University of Westminster, UNITED KINGDOM

## Abstract

**Introduction:**

Although the importance of training in patient safety has been acknowledged for over a decade, it remains under-utilized and under-valued in most countries. WHO developed the Multi-professional Patient Safety Curriculum Guide to provide schools with the requirements and tools for incorporating patient safety in education. It was field tested with 12 participating schools across the six WHO regions, to assess its effectiveness for teaching patient safety to undergraduate and graduate students in a global variety of settings.

**Methods:**

The evaluation used a combined prospective/retrospective design to generate formative information on the experiences of working with the Guide and summative information on the impacts of the Guide. Using stakeholder interviews and student surveys, data were gathered from each participating school at three times: the start of the field test (baseline), soon after each school started teaching, and soon after each school finished teaching.

**Results:**

Stakeholders interviewed were strongly positive about the Guide, noting that it emphasized universally important patient safety topics, was culturally appropriate for their countries, and gave credibility and created a focus on patient safety at their schools. Student perceptions and attitudes regarding patient safety improved substantially during the field test, and their knowledge of the topics they were taught doubled, from 10.7% to 20.8% of correct answers on the student survey.

**Discussion:**

This evaluation documented the effectiveness of the Curriculum Guide, for both ease of use by schools and its impacts on improving the patient safety knowledge of healthcare students. WHO should be well positioned to refine the contents of the Guide and move forward in encouraging broader use of the Guide globally for teaching patient safety.

## Introduction

Education of health-care professionals has given little attention to patient safety and the need to strengthen systems. Despite a plethora of patient safety initiatives at national and global levels, patient safety education for health professionals has not kept up with workforce requirements [1, 2].

Although the importance of education and training in patient safety has been acknowledged for over a decade [3], it remains under-utilized and under-valued in most countries. A UK study of the impact of teaching, learning and practicing patient safety in academic, organizational and practice contexts highlighted the need for patient safety education to be more explicit and better integrated into health care curricula [4].

The Commission on Education of Healthcare Professionals for the 21^st^ Century highlighted the weaknesses of health-care professional training, including: ‘…a mismatch of competencies to patient needs; curricular rigidities and static pedagogy; poor teamwork learning; narrow technical focus without broader contextual understanding; and weak leadership to improve health-system performance [5].’ The Commission recommended a system-wide reform to encourage adaptation, improvement and flexibility in health-care education generally, and to create a health-care professional workforce prepared for collaboration and trans- and inter-professional teamwork and able to adapt to local requirements and needs.

### The WHO Multi-professional Patient Safety Curriculum Guide

In 2011, WHO developed the WHO Patient Safety Curriculum Guide: Multi-professional Edition [6], which presents the general requirements necessary for incorporating patient safety in education. The Guide has the capacity to provide health-care professionals with the underpinning and applied knowledge that will facilitate the incorporation of patient safety principles into their practice, in a wide range of health-care delivery environments and health systems. It targets education in the fields of dentistry, medicine, midwifery, nursing and pharmacy, and other related health-care professions [6]. This Multi-professional edition is primarily used for implementing patient safety education in health-care universities/schools worldwide for educating undergraduate and postgraduate health-care professionals.

The Guide uses a health system-focused, team-dependent, and integrated approach to learning for health-care professionals and students. It contains information for all levels of faculty and staff, and it lays the foundation for capacity building in essential patient safety principles and concepts. The Guide was written to be applicable to different cultures and contexts using easily-understood language.

The development of the Multi-professional Curriculum Guide involved contributions by more than 50 international experts. The content development was guided by an Expert Working Group comprising experts from international professional associations in dentistry, medicine, midwifery, nursing and pharmacy, as well as from the WHO regions.

The Curriculum Guide consists of two parts. Part A is a teacher’s guide, which is designed to assist teachers to implement the Guide, laying the foundations for capacity-building in patient safety education. Part B provides a ready-to-teach, topic-based patient safety programme covering eleven patient safety topics, which can be implemented either as a whole or on a per topic basis.

The Curriculum Guide contents can be adapted and incorporated, in part or in whole, into existing university curricula, recognizing the variations in requirements of health-care professions' undergraduate curricula, context and resources.

### Evaluation Goal and Questions

The goal of the evaluation of the WHO Curriculum Guide was to assess the effectiveness of the Curriculum Guide as a resource for teaching patient safety to undergraduate and graduate students in a global variety of educational and cultural settings. The evaluation was designed to assess the performance of the Curriculum Guide focusing on: 1) the effectiveness of the Curriculum Guide as an educational resource to prepare health-care students for safe care; 2) the effectiveness of Part A of the Curriculum Guide in building capacity of educators/teachers in patient safety, as well as effects on students’ knowledge and attitudes about patient safety; and 3) the adaptability of the Curriculum Guide for universities with different cultures and social developmental contexts. Due to time and resource constraints, this evaluation study did not address the impact of the Curriculum Guide on translating knowledge into safe practice when it is still limited to examining experiences in the early use of the Curriculum Guide.

The following evaluation questions were addressed:

1. Does the Curriculum Guide contain the necessary and sufficient information and topics to allow its effective use in undergraduate training of health-care professionals?
2. What is the impact upon student learning of the inclusion of patient safety teaching in the curriculum?
3. In what ways can this Curriculum Guide be used to support the widespread implementation of explicit patient safety education globally?
4. How could the Curriculum Guide be modified in the future to best support teaching of patient safety to students in different environments?

## Methods

### Evaluation Design

**Fig 1:**
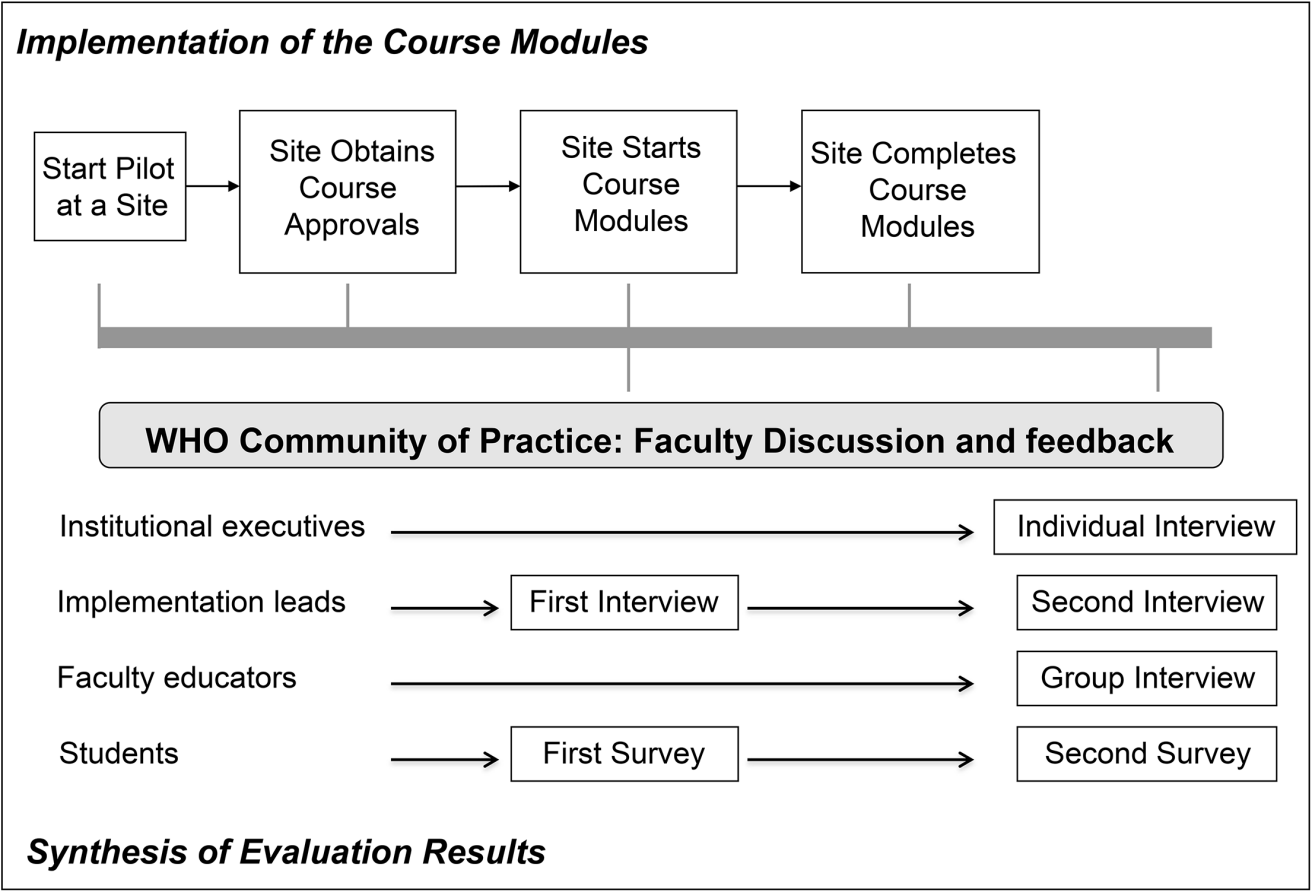
Diagram of the steps of the Curriculum Guide evaluation.

The evaluation of the Curriculum Guide is designed to generate formative information on the experiences of working with the Guide, for use in future implementation of the Guide, as well as summative information on the impacts of use of the Guide. Using a combined prospective/retrospective design, data were gathered on the experiences of universities/schools that participated in testing of the Curriculum Guide at three times: the start of the field test (baseline), soon after each school started teaching, and soon after each school finished teaching [7, 8]. The field test and evaluation timelines are shown in Fig 1.

This evaluation was designed to collect data from each of the following four stakeholder groups, recognizing that different groups of people may experience the same activity or programme quite differently [9]:

- Executives in the schools participating in the field test of the Curriculum Guide;
- Implementation leads–the individual at each school who facilitated the implementation of curricula based on the Curriculum Guide;
- Faculty educators who teach the patient safety courses;
- Students in the universities/schools who take the courses.

#### Formative Evaluation

The experiences of the universities/schools in using the Curriculum Guide were assessed, to provide feedback to WHO regarding capacity building, implementation issues and suggestions for improvements to the Guide, as well as to generate guidance for other universities/schools that might use the Guide in the future. The stakeholders’ perspectives are primarily those of the implementation leads and teaching faculty, who had the responsibility to spearhead the implementation of the Guide at each of their schools, and some data also were obtained from the student surveys. The implementation leads were interviewed at the beginning and the end of the course timelines, to allow us to identify changes in implementation experiences over time.

#### Summative Evaluation

The effectiveness of the Curriculum Guide was assessed using retrospective data from interviews and data from pre-and-post surveys of students taught the courses, as well as student survey data to assess changes in students’ patient safety knowledge and attitudes. The summative evaluation addressed all except one of the evaluation questions 3, which is addressed by the formative evaluation.

Following the external peer review process which considered the evaluation strategy, evaluation tools and research protocol by international experts, the proposal for the global evaluation of the Multi-professional Patient Safety Curriculum Guide was approved by the WHO Ethics Review Committee (ERC) in October 2011 (RPC 477). There was no deviation from the approved ERC proposal throughout the study. The implementation leads of each participating universities/schools ensured that local ethics approvals were in place in those countries where they are required prior to the initiating of testing.

The evaluation tools were either designed for this study, or had been previously developed and tested in the 2009–2010 evaluation study of the Patient Safety Curriculum Guide for Medical Schools [10]. These tools are available in the supplementary appendixes (S1 Table, S1 Text, S2 Text).

### Sample of Universities/Schools

Participation as a pilot site to the evaluation study is voluntary. A total of 12 universities/schools, two from each of the six WHO regions have been selected to ensure participating sites were recruited from Member States with different cultural and economic backgrounds, following WHO policies and through WHO channels to participate in the testing of the Curriculum Guide, which will be determined based on schools/universities volunteering and selection of those that meet evaluation criteria approved by the WHO ERC (S3 Text). The universities and schools that participated in the Curriculum Guide evaluation were identified by WHO by the end of June 2011. The written agreements from each of 12 participating universities/schools in Table 1 were received to secure their commitment in this field study. Each school was asked to select at least four patient safety topics they planned to teach using the Guide, allowing flexibility in how they integrated these courses into their overall curricula.

**Table 1:** Universities/Schools that participated in the Curriculum Guide field test and evaluation.

Region and	Disciplines of Students Taught Using the Guide			
University/School	Nursing Midwifery Dentistry Pharmacy			
**African region** E**thiopia: Gondar University** Z**imbabwe: University of Zimbabwe**	X		X	
**Eastern Mediterranean region**				
Egypt: Cairo University			X	
Jordan: University for Sciences and Technology	X	X		
**European region**				
Greece: University of Athens			X	
UK: University of the West of Scotland	X	X		
**Pan American region**				
Argentina: University of Del Salvador	X			
Mexico: National Autonomous University of Mexico			X	
**South East Asian region**				
India: All India Institute of Medical Sciences (AIIMS)			X	
Sri Lanka: University of Peradeniya	X			
**Western-Pacific region**				
The Philippines: University of the Philippines	X	X		
Malaysia: United Nations University / Universiti Kebangsaan Malaysia	X			X
**Total Sites**	7	3	5	1

A total of 14 schools, two or three from each of the six WHO regions, were identified. Two sites, one from the European region and one from the Western-Pacific region, withdrew due to academic scheduling difficulties. The 12 schools that completed the field test were evenly distributed geographically, with two schools located in each of the six WHO Regions (Table 1).

### Data Collection

Participating universities/schools commenced teaching their selected topics sequentially from September 2011, each teaching on different timelines. The evaluation timeline at each university/school was linked to when it commenced teaching its students, with data collection at each participating university/school starting as close as possible to the first week of teaching the courses, and ending within two months after course completion. This approach allowed collection of consistent data relative to the teaching timelines of the participating universities/schools.

Two data collection methods were used (Fig 1). The first was telephone interviews with each of three key stakeholder groups: the implementation leads, teaching faculty, and executives at the participating universities/schools. The second was pre-teaching and post-teaching surveys of the students taught the patient safety topics at those sites.

#### Stakeholder interviews

We developed written interview protocols (S1 Table, S1 Text) that guided the interviews with the three stakeholder groups and also served as the frameworks for recording the interview notes in a consistent format. We first developed a master list of questions to be used in the interviews; then we created a matrix that identified which of the listed questions would be asked of each of the stakeholder groups. Using this matrix, a separate protocol was prepared for interviews with each stakeholder group. For the implementation leads, two protocols were prepared–one for interviews conducted early in the implementation process and the other for interviews conducted after course completion.

All the stakeholder interviews were conducted by telephone, with the exception of four that were received as written comments, due to limited availability of respondents and some language problems. The relevant interview protocol was shared with the respondents before they were interviewed. Written informed consent (S4 Text) was received from each respondent prior to conducting the interview.

One interviewer conducted each interview following the interview protocols. The interviews were recorded, and the interviewers used the recording to prepare the written notes from the interviews. They entered the data into the interview protocol format, and then shared the notes with the respondents for comments and feedback, with revisions to the notes as needed.

#### Student surveys

We developed two questionnaires for the student surveys (S2 Text): a pre-teaching and a post-teaching questionnaire. Both questionnaires contained identical items for topics related to (1) students’ perceptions and attitudes about patient safety and (2) their knowledge of patient safety facts and processes, to allow pre-post comparisons. The post-teaching questionnaire also contained a third set of questions that obtained students’ feedback on the patient safety topics they had been taught.

The survey section on students’ perceptions and attitudes contained 23 questions, which were grouped in four domains: patient safety knowledge, health-care system safety, personal influence over safety, and personal attitudes about safety. These questions were drawn from a student survey used in the evaluation of the WHO Curriculum Guide for Medical Schools [10].

For the patient safety knowledge questions, two to four questions were written for each of the patient safety topics in the Curriculum Guide. The questions, and the correct answers to them, were developed by WHO staff and reviewed by the lead author of the Curriculum Guide and the WHO patient safety education team. The student surveys at each site were tailored to contain the knowledge questions only for the topics that the school had selected to teach.

The student feedback questions on the post-teaching survey consisted of 16 questions in two domains: effectiveness of the topics (eight questions) and effectiveness of teaching (eight questions). These questions also were drawn from a student survey for the evaluation of the Curriculum Guide for Medical School [10], with some additional questions written by the WHO patient safety education team.

All the students being taught the Curriculum Guide topics were asked to complete the pre-teaching and post-teaching surveys. Written informed consent (S4 Text) was obtained from each student participating in this study, and additional informed consent language was provided at the start of each survey, which included a statement that they could choose not to complete the survey if they so wished.

The participating universities/schools administered the student surveys to their students, following instructions provided by the WHO team. The schools then sent the completed student surveys to the WHO team for data entry.

A team of five WHO personnel entered the data from the student surveys into excel spreadsheets using a standard template for entry cells and data coding. Data entry personnel were trained in these methods and participated in debriefings to ensure data entry consistency. The data were audited to check completeness of survey entries, avoid duplicate entries, and check the accuracy of the data entered.

The total sample for the pre-teaching surveys was 1,410 students in the 12 participating schools; the total sample for the post-teaching survey was 1,036 students. The distributions of students by region, school, and discipline differed slightly for the pre-teaching and post-teaching surveys, but the samples were consistent enough to allow for valid analysis of changes in students’ perceptions and knowledge between these two times.

### Data Analysis

A combination of qualitative and quantitative methods were used to analyze the data collected in the evaluation. The central organizing structure for the analysis was the set of four questions to be answered by the evaluation. For the formative evaluation, a content analysis was performed of interview responses from the implementation leads about the experiences of their schools in implementing the Curriculum Guide [11, 12]. For the summative evaluation, content analysis of qualitative data and quantitative analysis of survey data were used together to assess effectiveness of the Curriculum Guide from the perspectives of different stakeholder groups. As described above, many of the same interview questions were asked of more than one stakeholder group, and we compared their responses during the analysis of the interview data. The content analysis identified common themes and variations in responses among the stakeholders. For the implementation leads, responses at the start and end of their teaching periods were compared to identify any changes in their views during the course of the field test regarding their implementation experiences.

The analysis of changes in students’ perceptions and knowledge of patient safety were performed as comparisons of two cross-sectional samples of students–for the pre-and post-teaching surveys. The information of interest was the extent to which aggregate changes occurred in students’ perceptions and knowledge of the topics they were taught. Pre-post differences in values were tested for statistical significance.

#### Perceptions and attitudes

For each respondent, a composite variable was created for each of the four domains of patient safety knowledge, health-care system safety, personal influence over safety, and personal attitudes about safety. Each composite variable was calculated as the mean of the scores for the survey questions within the domain (on the 1-to-5 scale used in the individual survey questions). These four domains, and the items in them, have a face validity regarding the relevance of each item to its domain. We tested these relationships empirically, finding that the items contained in each domain are much more highly correlated with their domain composite variables than they are with any of the other three domains.

A new dichotomous variable, called “top response,” was generated for each survey question response, for which top response = 1 if the original response was either 4 or 5 on the 5-point scale, or otherwise top response = 0. A top response score also was generated for each student’s newly created domain composite scores. We analyzed these measures by comparing the means for the before-and-after scores.

#### Patient safety knowledge

Different sets of patient safety knowledge questions were relevant for students at each participating university/school. Thus, the measures used to assess changes in knowledge were the percentages of the relevant questions that each student answered correctly. These percentages were calculated for each patient safety topic and also for the aggregate of all topics that were taught in each student’s school. We compared percentages of correct answers given on the pre-teaching and post-teaching surveys.

## Results

### A. Integrity of the Curriculum Guide

To answer the first research question on implementation, feedbacks on the effectiveness of the Curriculum Guide were presented in both stakeholders’ interviews and students’ post-teaching surveys.

The stakeholders interviewed were positive, overall, regarding the effectiveness of the Curriculum Guide in supporting their patient safety teaching efforts. Several sites observed that the Guide gives credibility and creates a focus on patient safety, bringing the subject to the eye of the academic community. They felt that the Guide emphasizes universally important patient safety topics, including for their countries, and it shows how to organize them for teaching. All the sites reported that the Guide contents are culturally appropriate for their countries. Several of them noted that they adjusted some of the case studies and other contents to make them more applicable to their situations, and that the Guide was readily adaptable.

The sites generally reported that the Guide was easy to follow, user-friendly in a format that was nicely presented, and readily adaptable to their learning outcomes. The faculty reported that use of English was easy for most of them to understand, although there was a request for translation of the Guide into Spanish.

The majority of the sites found that Part A of the Guide enabled them to develop the skills and knowledge base of teaching faculty, for capacity building. They also noted, however, that it would take time to fully develop the capability to teach patient safety effectively. More mixed reactions were found regarding the teaching tools provided in Part B, noting some difficulties in working with some of them, such as difficulties in using articles and references provided in the Guide. The two tools of greatest value to the faculties were the teaching slides and the case studies.

The following are the specific comments highlighted by respondents during interviews:

1. The WHO Curriculum Guide has been very helpful to teach patient safety because it brings all the key patient safety topics together in one place. Before we had it, many schools took a more piecemeal approach when they thought the topic was necessary.
2. The curriculum guide has clear presentation of information and guidance for educators to give directly to the students, although these need to be customized by adding local stories, case studies, and examples that attract the attention of the students.

In the post-teaching student survey, 82.8% of the students gave the teaching (Part A) a 4 or 5 score, averaged across eight items in the domain. The students also reported that the contents of the patient safety topics (Part B) were highly effective, with 93.3% of the students giving the topics a 4 or 5 score, averaged across the eight items in the domain (Table 2).

**Table 2:** Percentage of top responses on student perceptions about teaching effectiveness and patient safety topics taught, post-teaching survey.^+^

	Number of Responses	Percentage of Top	95% Confidence Interval	
			Lower	Upper
**Effectiveness of Teaching**				
Total teaching effectiveness	495	82.8	79.5	86.2
Teaching style helped learning	785	78.2	75.3	81.1
Instructors helped understanding	783	85.2	82.7	87.7
Culturally appropriate presentation	775	83.6	81.0	86.2
Teaching aids added to session	726	76.2	73.1	79.3
Assignments helped understanding	526	69.2	65.2	73.2
Sufficient time for topic	776	70.0	66.7	73.2
Assessment methods effective	748	71.8	68.6	75.0
Appropriate time in curriculum	737	75.7	72.6	78.8
**Effectiveness of Patient Safety Topics**				
Total topics effectiveness	760	93.3	91.5	95.1
Aims of topic were clear to me	795	86.9	84.6	89.3
Patient safety training in curricula	796	90.2	88.1	92.3
Improved my knowledge/skills	794	86.6	84.3	89.0
Acquired new knowledge/skills	795	88.1	85.8	90.3
Able to apply knowledge taught	794	84.6	82.1	87.1
Understand more of importance	799	89.6	87.5	91.7
More knowledge of practices	783	85.1	82.6	92.2
Training increased my motivation	779	90.1	88.0	92.2

**+** Top responses = responses of either 4 or 5 on a 5-point scale.

### B. Impact on student patient safety attitudes and knowledge

#### Perceptions and attitudes

Substantial and statistically significant changes were observed in the perceptions and attitudes of students regarding patient safety, from baseline to post-teaching of the topics (Table 3). For the first of the four domains–knowledge of patient safety–the percentage of students giving top responses (4 or 5 on the scale) increased from 19.2% to 56.3% (p<0.001) for the domain, with large increases (p<0.001) for each of its seven items. Similar results were found for two of the other domains: increases in top responses from 28.0% to 41.0% (p< 0.001) for health-care system safety and from 28.0% to 55.9% (p< 0.001) for personal influence over safety.

**Table 3:** Percentage of top responses by students on their knowledge and attitudes of patient safety, pre and post teaching.^+^ ^,^ ^#^

	Pre-Teaching		Post-Teaching	
	Percentage	Std Dev	Percentage	Std Dev
**Patient Safety Knowledge**				
Total knowledge domain	19.2 ***	39.4	56.3 ***	49.6
Types of error in health care	20.3 ***	40.3	51.1 ***	50.0
Factors contributing to error	28.9 ***	45.3	57.2 ***	49.5
Factors influencing patient safety	35.4 ***	47.8	62.5 ***	48.4
Ways to speak up about error	19.1 ***	39.2	47.1 ***	49.9
What should do if error made	27.0 ***	44.4	57.1 ***	49.5
How to report an error	21.4 ***	41.0	49.2 ***	50.0
Role of organization in reporting	23.8 ***	42.6	52.5 ***	50.0
**Health-care System Safety**				
Total system safety domain	28.0 ***	44.9	41.0 ***	51.8
Health-care workers make errors	41.9 ***	49.4	56.4 ***	49.6
My country has safe health system	37.7 ***	48.5	45.0 ***	49.8
Medical error is common	48.3 ***	48.5	56.6 ***	49.6
Unusual give patients wrong drug	35.8	48.0	37.2	48.4
Staff get patient safety training	56.4	49.6	59.8	51.8
**Personal Influence Over Safety**				
Total personal influence domain	38.0 ***	48.6	55.9 ***	49.7
Easy to tell others of my error	42.3 **	49.4	49.0 **	50.0
Easier to find someone to blame	34.1	47.4	38.1	48.6
Confident to speak to someone	50.5 ***	50.0	61.5 ***	48.7
Know talk to people who erred	39.8 ***	49.0	57.4 ***	49.5
Able to ensure safety is good	45.4 ***	49.8	55.8 ***	49.7
Believe reporting will help safety	75.8 *	42.8	80.0 *	40.0
Able to talk about own errors	68.8 ***	46.3	77.1 ***	42.0
**Personal Attitudes of Safety**				
Total attitudes of safety domain	93.9	23.9	95.3	21.2
Can contribute by knowing causes	84.0 **	36.7	88.0 **	32.5
Learn from mistakes to improve	87.0 **	33.6	89.5 **	30.6
Deal with my errors part of job	88.3 **	32.1	92.1 **	26.9
Learn to deal with errors in training	90.7	29.0	92.6	26.2

**+** Top responses = responses of either 4 or 5 on a 5-point scale

* p < 0.05

** p < 0.01

*** p < 0.001

# For a few of the measures, the significance of pre-post differences in scores differed for the top responses versus original 5-point scales, which reflected the distributions of responses. In some cases, changes in perceptions appeared to be larger at the lower values of the 5-point scale (a phenomenon of bringing up the bottom scores). These differences, however, did not affect the overall evaluation results.

For the domain of personal attitudes of safety, 93.9% of students gave top responses at baseline, and the percentage did not increase significantly in the post-teaching survey. Small increases were found for three of its individual items (p< 0.01).

#### Patient safety knowledge

WHO received feedback from the participating universities/schools that the patient safety knowledge questions on the student survey were quite difficult. This difficulty is reflected in the low percentages of these questions that students answered correctly in the pre-teaching survey. In the post-teaching survey, however, the percentage of correct answers doubled overall (from 10.7% to 20.8%) from the pre-teaching survey, and even larger increases were found for some of the specific patient safety topics (Table 4). Students showed the largest improvement in knowledge for the two topics of *infection control* (from 11.9% to 46.9% correct) and *invasive procedures* (from 15.6% to 38.1% correct). Student knowledge appeared to decline, however, for the topic *learning from error* (from 12.1% to 7.1% correct).

**Table 4:** Differences in Students Knowledge of Patient Safety, Before and After Teaching.

	Pre-Teaching		Post-Teaching	
Topic	Number	Mean Percent +	Number	Mean Percent +
1. Patient Safety *	508	11.5	424	22.4
2. Human Factors *	45	1.5	116	9.2
3. Systems Effects *	37	5.4	19	29.8
4. Teamwork *	324	10.2	176	20.6
5. Learn from Error *	226	12.1	156	7.1
6. Clinical Risk	138	30.0	131	27.7
7. QI Methods #		NA		NA
8. Engage Patients *	103	0.5	74	10.1
9. Infection Control *	268	11.9	179	46.9
10. Invasive Procedures *	307	15.6	231	38.1
11. Medication Safety *	325	7.7	290	14.1
All possible answers *	1,074	10.7	904	20.8

+ The mean percent is the mean of the percentage of possible answers that each student answered correctly, measured for each topic in the Curriculum Guide and overall for all possible topics (those taught by each school).

# No data were available for Quality Improvement Methods (Topic 7) because it was not taught at any of the participating schools.

* Statistically significance difference (at p < 0.05 level) in mean percent correct answers between pre-teaching and post-teaching student surveys, as measured by comparisons of the 95% confidence interval thresholds. Improvement is significant when the lower threshold for post-teaching survey is higher than the upper threshold for the pre-teaching survey (i.e., the two confidence intervals do not overlap).

#### Faculty perspectives regarding impacts on student learning

Although the teaching faculty were enthusiastic about the value that the Curriculum Guide offers them, they were cautious about estimating the early impacts of the Guide on the patient safety practices of the students they taught. They believed that their students’ knowledge of patient safety issues and practices has grown, but many of them stated that it was too early to assess the impact of that knowledge on the students’ subsequent practices.

### C. Use of Curriculum Guide for global implementation of patient safety education

The majority of the sites reported that use of the Curriculum Guide offered good value and was clearly a positive educational investment. As one school executive stated: “with proper training, the academics will be equipped with standardized knowledge to teach the students based on the information provided in the Guide.” The sites noted their concerns about the widespread lack of knowledge about patient safety in health-care organizations. Given their successful experiences with the Curriculum Guide, they felt that use of the Guide should be expanded globally to improve the safety of health-care practices. Several of them already had begun outreach to share their experiences with others and encourage them to teach patient safety using the Guide.

One of the greatest successes reported by the sites was the extremely positive reception by their students to the patient safety training and the substantial benefits to the students. Implementation challenges included the difficulty of changing culture to be patient safety oriented, lack of knowledge of faculty members about patient safety, designing and implementing the teaching, student reactions, and achieving sustainability of what the students learned. They also offered suggestions for improving the Guide based on their experiences working with it.

Sites’ choices for the topics to teach were based on relevancy to the needs of their students, the capabilities of the schools, and what students already were being taught. Topic 7 was not taught at any of the participating schools, and one school clearly stated that it was not selected because they did not have faculty with sufficient knowledge in this field. Many sites had a goal to eventually teach all eleven topics in the Guide and to integrate them appropriately within the larger curricula.

### D. Improvements to the Curriculum Guide

To identify opportunities to improve the contents of the Curriculum Guide, the stakeholders interviewed were asked to provide feedback on its usability, and its strengths and weaknesses. Then they were asked for suggestions on how the Guide might be improved, including general suggestions as well as suggestions for improvements specifically to Part A and Part B of the Guide. Students were asked in their post-teaching survey to give feedback or suggestions for improvement.

In general, the sites had a positive response to the Guide, and they highlighted its greatest strengths to its comprehensiveness, effective organization, and patient safety topics addressed. For weaknesses identified, the focus tended to be on the need to adapt the contents to local situations and specialties. Some other concerns about the Curriculum Guide involved “… it may be difficult to apply the Guide to teach patient safety in clinics and other resource-limited settings.”

A number of suggestions for improvements were offered. For the Part A, these included introducing new teaching methods and materials, expansion of guidance for training educators on patient safety, adaptation of some contents for use in clinical care settings. For the Part B, while no site stated that any topics should be deleted from the Curriculum Guide, but some had suggestions for changes in emphasis or reorganization of topics. Numerous suggestions were offered for addition of new topics, such as an introductory topic that addresses overall patient safety issues, patient safety relevant to specific types of clinical care, and several aspects of patient safety at the leadership and organizational level.

## Discussion

The evaluation of the WHO Multi-professional Patient Safety Curriculum Guide gathered information from 12 universities/schools that participated in this field test. The evaluation yielded positive findings regarding both the contents of the Guide and the experiences of the faculty and students in teaching its contents at their schools. In addition, valuable insights were shared by the participating schools about possible modifications to strengthen the Curriculum Guide and enhance its usefulness for schools that use it.

The key evaluation results are summarized to answer the four questions directly.

### A. Does the Curriculum Guide contain the necessary and sufficient information and topics to allow its effective use in undergraduate training of health-care professionals?

According to the participating universities/schools, the Curriculum Guide contains the information they need to use it effectively in patient safety training their students. They received the guide enthusiastically because it provides a comprehensive, structured package that schools can use easily to teach patient safety. The topics in the guide are relevant to the patient safety issues in their countries; many sites noted “…these issues are universal in almost all Member States”. The contents of the guide also are compatible with the cultures of the Member States and schools.

It is necessary and desirable for schools to adapt the guide contents to their local situations. The participating schools did this by developing examples and case studies that are relevant to their local situations, which are more able to get the attention of students and reinforce their learning.

The dentistry schools had the greatest trouble with the guide because the Guide contents are aimed at medical care and in-hospital settings, which are very different from community-based dental practices. Therefore more modifications are needed for the dentistry schools to make the Curriculum Guide more relevant to dentistry.

### B. What is the impact upon student learning of the inclusion of patient safety teaching in the curriculum?

The teaching of patient safety by the participating universities/schools was found to substantially strengthen students’ understanding of patient safety. At the start of the field test, only 20.4% of the students reported they had previously had a patient safety course, and the percentages ranged from a low of 1.8% to a high of 73.7% at individual schools. For many of the students, therefore, this was the first time they received focused training in these patient safety topics.

After taking the courses, the students’ knowledge of the patient safety topics they were taught showed substantial and statistically significant improvements, although it is not clear how well statistical significance related to clinical significance due to the study design. The percentage of correct answers doubled overall (from 10.7% to 20.8%) between the pre-teaching and post-teaching surveys, though were still low due to the difficulty of the questions. The largest improvement found was for the topics of infection control (from 11.9% to 46.9% correct) and invasive procedures (from 15.6% to 38.1% correct).

The training also elevated students’ perceptions and attitudes toward the importance of patient safety and their ability to influence it. The scores the students gave for three of the domains for perception/attitudes (patient safety knowledge, health-care system safety, personal influence over safety) increased substantially. The lack of change in the fourth domain (personal attitudes domain of safety) was likely because these ratings already were high at baseline and had little space for further increase.

### C. In what ways can this Curriculum Guide be used to support the widespread implementation of explicit patient safety education globally?

The experiences of the participating universities/schools in implementing the Guide can inform strategies to move toward global implementation of patient safety education. Before introduction to the Guide, many of the participating schools had not defined patient safety as a priority, but as they worked with the Guide, their commitments quickly grew. The teaching faculties at many schools were found to have limited patient safety knowledge, suggesting that faculty may need more extensive training than the schools originally had expected, to prepare them well to teach these topics. The schools emphasized that this experience was just a start; that it will take several years before they achieve the full scope and quality of patient safety teaching they desire.

Most faculties were enthusiastic, and those who resisted initially were often motivated by workload issues. Integrating the guide into existing curricula helped schools to deal with workload issues, but it lowers the visibility of patient safety. Therefore, in the long run, some schools prefer to teach it as separate course(s). All the sites taught only a few topics, but many of them plan to expand in the future to eventually teach all eleven topics.

The participating schools identified lists of successes and challenges they experienced in implementing the Guide. They also described new strategies they wish to pursue to expand use of the Guide to teach patient safety. By applying these lessons to further work with the Guide, there is strong potential for successful use of the Guide in teaching patient safety globally.

### D. How could the Curriculum Guide be modified in the future to best support teaching of patient safety to students in different environments?

There was virtually unanimous agreement that the Guide’s greatest strength is its comprehensiveness, while its most frequently cited weakness is its adaptation. Using English for the Curriculum Guide would be found difficult for its acceptance in some countries.

Specific suggestions were provided for improvement for both the Part A and Part B of the Curriculum Guide, which generates guidance for other schools that could be using the Curriculum Guide in the future.

This study had a number of limitations due in part to the worldwide nature of the project. The tight timescale and resources did not allow detailed testing of all evaluation tools prior to the study. Only 12 universities/schools were able to participate in this study, which could affect the generalizability of the results, and the sample of each location is not sufficient to allow comparison between different location and professions.

In addition, the risk of self-report bias, which always is a consideration in interview or survey work, was reduced by findings of strong consistency in responses to questions across multiple stakeholder groups. Although teaching slides for each topic were provided to all participating sites, they were allowed to deliver the selected topics using their usual teaching approaches with local available resources. Nevertheless, the integrity of the data generated in the evaluation is estimated to be quite high because of both the design of the data collection instruments and the well coordination in collecting and coding of data from interviews and the student surveys, resulting in a high completeness of the data. The quality of the collection and coding of survey data was protected by provision of clear instructions to the sites on how to administer the survey, on-going support to the sites by the WHO staff, and coding of survey results by the WHO staff using structured coding schemes.

In conclusion, this evaluation has generated positive results regarding the effectiveness of the Curriculum Guide, its impacts on improving the patient safety knowledge of healthcare students, and additional information generated by the field test and evaluation. Using these findings, WHO should be well positioned to refine the contents of the Curriculum Guide and to move forward in encouraging use of the Guide globally by organizations to teach patient safety.

## Supporting Information

S1 Table Master List of Questions Used in the Stakeholder Interviews. (PDF)

S1 Text Interview Protocols. (PDF)

S2 Text Questionnaire for Post-Teaching Student Survey. (PDF)

S3 Text Criteria for Selecting Participating Pilot Sites. (PDF)

S4 Text Informed Consent Forms. (PDF)
