# Supplementary material for: Field Test of the World Health Organization Multi-Professional Patient Safety Curriculum Guide
Source: PLoS One. 2015 Sep 25;10(9):e0138510. doi: 10.1371/journal.pone.0138510 (PMC4583458; doi:10.1371/journal.pone.0138510)
Supplement: S1 Table — (PDF) [file pone.0138510.s001.pdf]

# S1 Table. Master List of Questions Used in the Stakeholder Interviews

| Topics and Questions | Implementation Coordinator Pre-teaching | Implementation Coordinator Post-teaching | Institution Executive | Teaching Faculty |
|---|---|---|---|---|
| Contents of the Curriculum | | | | |
| *Topics and Approach* | | | | |
| Is patient safety already being taught in your school? | X | | | |
| How does the Curriculum Guide inform your organization about the key topics in patient safety? | X | X | X | X |
| How helpful is the Curriculum Guide for educators to develop capacity and skills to teach patient safety? | X | | | |
| Is the mix of topics in the Curriculum Guide consistent with their relative importance as safety issues in your country? | X | X | X | X |
| What additional topics should be addressed in the Curriculum Guide? | X | | | X |
| What topics should be deleted or reduced in importance? | X | | | X |
| What suggestions do you have to improve the Patient Safety Topics in the Curriculum Guide? | | X | X | X |
| *Support for Educators (Part A)* | | | | |
| Overall, how helpful is the Curriculum Guide for educators to develop capacity and skills to teach patient safety? | | X | X | X |
| How effective is the Curriculum Guide in assisting educators to integrate patient safety learning into their curricula? | | X | | X |
| How effective is the Curriculum Guide in informing educators on: | | | | X |
| Importance of building supportive environments for teaching patient safety | | | | |
| How to design and implement patient safety curricula | | | | |

| Topics and Questions | Implementation Coordinator Pre-teaching | Implementation Coordinator Post-teaching | Institution Executive | Teaching Faculty |
|---|---|---|---|---|
| Using inter-disciplinary teaching in patient safety | | | | |
| How culturally appropriate for your country are the contents of the Curriculum Guide? (methods, skills and knowledge) | | X | X | X |
| How appropriate are the assessment strategies provided in the curriculum guide for the resources and teaching environment in your country? | | | | X |
| How appropriate are the evaluation strategies in the Curriculum Guide for evaluating the patient safety courses | | | | |
| What suggestions do you have to improve the Teacher's Guide? | | X | | X |
| *Course Content (Part B)* | | | | |
| How helpful to you was the overall content of the Curriculum Guide topics in teaching patient safety to your students? | | X | | X |
| How helpful are tools/resources provided for teaching patient safety?<br>Learning objectives<br>Knowledge requirements<br>Performance requirements<br>Teaching slides<br>Patient safety resources | | | | X |
| Experiences Implementing Curriculum Guide | | | | |
| Which Curriculum Guide topics were taught at your school? Why were these topics chosen? | X | X | X | |
| How were decisions made to use the Curriculum Guide for teaching patient safety and for choices of specific topics? | X | | X | |
| Who was involved in making these decisions and how were they involved? | X | | | |

| Topics and Questions | Implementation Coordinator Pre-teaching | Implementation Coordinator Post-teaching | Institution Executive | Teaching Faculty |
|---|---|---|---|---|
| *Probe*: institutional leadership, faculty, students, others<br>*Probe*: seminars, meetings, focus groups, surveys of faculty | | | | |
| What priority did the institution's leadership give to this patient safety teaching? | | | X | |
| What was your experience in obtaining agreements and approvals to teach the topics as part of the educational curriculum? | X | | | |
| How did you introduce the selected patient safety topics? Were they taught as separate course(s), or were they integrated into existing curricula? | | X | | |
| What priority did the institution's faculty give to this patient safety teaching? | | | | X |
| How did you integrate teachings of patient safety topics across different clinical disciplines? | | | | X |
| Were local experiences, case studies, or literature incorporated in teaching patient safety topics? | | | | X |
| What methods did you use to assess students' learning? | | | | X |
| What were your greatest successes in introducing the topics? | | X | | X |
| What were your greatest challenges in introducing the topics ? | | X | | X |
| What changes did you make during the training to address issues that arose during the implementation of the Curriculum Guide topics? | | X | | X |
| Based on your experience with teaching these topics, how might you change your approach to teaching them in the future? | | X | | X |
| How did faculty respond to evaluation of the patient safety topics introduced? | | X | | X |
| Usability of the Curriculum | | | | |
| How readily could you integrate the contents of each topic with actual practices and issues involved in professionals' care delivery roles? | | | | |

| Topics and Questions | Implementation Coordinator Pre-teaching | Implementation Coordinator Post-teaching | Institution Executive | Teaching Faculty |
|---|---|---|---|---|
| Are you able to adapt the contents of the Curriculum Guide topics easily to local cultural needs and requirements? | | | | X |
| How sufficient was the evidence-base presented in the topics? | | | | |
| How well did the content of the topics help students understand how they could use what they learned in future professional practices/actions? | | | | |
| How easy was it to introduce the Curriculum Guide topics selected into existing curricula? | | X | X | |
| Which tools did you use? For those not used, why did you not use them? | | | | X |
| How easy is the language which theCurriculum Guide is written:<br>for educators? for students? | | X | X | X |
| Are the Curriculum Guide topics presented in a user-friendly format? | | X | | X |
| Acceptance of the Curriculum Guide | | | | |
| What was the response of colleagues and other educators to the Curriculum Guide? How supportive were they of teaching the Patient Safety topics? | | X | X | |
| For faculty who were critical of the Curriculum Guide, what were the reasons for their criticisms? | | X | | |
| What did the faculty see as the strengths of the Curriculum Guide? | | | | X |
| What did the faculty see as the weaknesses of the Curriculum Guide? | | | | X |
| Value of Training Provided | | | | |
| What value does the Curriculum Guide contribute to the 'knowledge base' required by students for their professional training? | | X | X | X |
| Have the patient safety courses had an impact on current student clinical performance in terms of practicing safe care? | | | | X |
| For advancing patient safety knowledge and practice, which of the topics of the Curriculum Guide content are most valuable? | | X | | |

| Topics and Questions | Implementation Coordinator Pre-teaching | Implementation Coordinator Post-teaching | Institution Executive | Teaching Faculty |
|---|---|---|---|---|
| What of the Curriculum Guide topics are the least valuable? | | X | | |
| How important an educational investment is the Curriculum Guide, considering the knowledge value it offers and the resources involved in teaching it? | | X | X | |
| Effects on the Institution's Patient Safety Capacity | | | | |
| Before using the Curriculum Guide, how much had the institution included patient safety topics in its professional training curricula? | | X | X | X |
| How much does the Curriculum Guide contribute to strengthening the institution's capacity to teach about patient safety issues and practices? | | X | X | |
| What additional capacity would the institution like or need to have to strengthen its patient safety capacity further? | | X | X | X |
| Opportunities to Improve the Curriculum Guide | | | | |
| What changes should be made to strengthen the Curriculum guide? | | | X | X |
| Other Potential Applications for the Curriculum | | | | |
| In what other ways would you like to use the Curriculum Guide at your institution? | | X | X | |
| How can the Curriculum Guide be used by organizations other than universities or schools that train health-care professionals? Which types of organizations? | | | X | X |
| What advice about how best to apply the Curriculum Guide would you give to other schools regarding use of the Curriculum Guide? | | X | | X |
