# Supplementary material for: Field Test of the World Health Organization Multi-Professional Patient Safety Curriculum Guide
Source: PLoS One. 2015 Sep 25;10(9):e0138510. doi: 10.1371/journal.pone.0138510 (PMC4583458; doi:10.1371/journal.pone.0138510)
Supplement: S1 Text — (PDF) [file pone.0138510.s002.pdf]

S1 Text. Interview Protocols

Protocol for Institutional Executive Interviews

Evaluation of the WHO Patient Safety Curriculum Guide, Multi-professional Edition

The following questions will inform the evaluation:

a. Does the curriculum guide contain relevant information and appropriate patient safety topics for inclusion in undergraduate training of health professionals?
b. In your view how did the students respond to patient safety teaching included in the curriculum?
c. Can you suggest ways that this Curriculum Guide be used to support the widespread implementation of explicit patient safety education globally?
d. How could the Curriculum Guide be modified in the future to best support teaching of patient safety to students in different environments?

Privacy and Confidentiality

Participation in the evaluation will be voluntary. All interviews will be conducted at times convenient to the participants, and every step will be taken to accommodate reasonable times to conduct the interview.

All data will be collected, stored and managed with strict confidentiality. Stringent safeguards will be taken to ensure confidentiality and anonymity for all interview/group discussion data across schools/universities. A secure WHO telephone line will be used to conduct all interviews. Interview data that is gathered from '*Institutional executives*' will be securely stored in password protected computers.

Written consent will be sought from all stakeholders prior to the start of interviews or discussion groups. Permission will also be sought to allow taking minutes from the interviews/group discussions. If a participant declines permission, his/her interview will not be recorded. Interviewees will be provided a Word file containing a summary of their interviews and offered the chance to comment and verify that the log is accurate.

All logs from the interviews will be de-identified. No interview data will be listed by name and no comparison will be made across schools/universities.

Interview Questions

1. Contents of the Curriculum Guide

<u>1.1 Topics and Approach</u>

Did the Curriculum Guide inform your organization about the key topics in patient safety?

Does the range of topics in the Curriculum Guide reflect the relative importance of safety issues in your country?

Do you have any suggestions that to improve the Patient Safety Topics in the Curriculum Guide?

1.2 Support for Educators (Part A)

Overall, how helpful is the Curriculum Guide for educators to develop capacity and skills to teach patient safety?

How culturally appropriate for your country are the contents of the Curriculum Guide? (methods, skills and knowledge)

2. Experiences Implementing Curriculum Guide

Is patient safety already being taught in your school?

Which Curriculum Guide topics are already being taught at your school? Why were these topics chosen?

How were decisions made to use the Curriculum Guide for teaching patient safety and for choices of specific topics?

What priority did the institution's leadership give to this initiative in patient safety teaching?

What priority did the institution's faculty give to the WHO initiative in patient safety teaching?

How did faculty respond to evaluation of the patient safety topics introduced?

3. Usability of the Curriculum

How easy was it to introduce the Curriculum Guide topics selected into the Faculty's existing curricula?

How easy or difficult to understand is the language used in which the Curriculum Guide n:

for educators? for students?

4. Acceptance of the Curriculum Guide

What if any was the response of colleagues and other educators to the Curriculum Guide? Were colleagues supportive of teaching the Patient Safety topics?

5. Value of Training Provided

Did the Curriculum Guide contribute to the knowledge base students require during their professional training?

Which topics in the Curriculum Guide advance patient safety knowledge and practice?

Which topics in the Curriculum Guide are the least valuable?

Is the Curriculum Guide a sound investment considering the knowledge value it offers and the resources involved in teaching it?

6. Effects on the Institution's Patient Safety Capacity

Before using the Curriculum Guide, did your institution include patient safety topics in the curricula?

How much does the Curriculum Guide contribute to strengthening the institution's capacity to teach patient safety?

What additional capacity would the institution like or need in order to strengthen its patient safety capacity?

7. Opportunities to Improve the Curriculum Guide

What do you think are the strongest parts of the Curriculum Guide?

What do you think are the weakest parts of the Curriculum Guide?

What changes would you suggest to improve the weak parts identified?

8. Other Potential Applications for the Curriculum

In what other ways do you think you could use the Curriculum Guide at your institution?

Do you think organizations other than universities or schools that train health care professionals might find the Curriculum Guide useful? If so which types of organizations?

Protocol for Implementation Coordinator Interviews

Evaluation of the WHO Patient Safety Curriculum Guide, Multi-professional Edition

The following questions will inform the evaluation:

a. Does the curriculum guide contain relevant information and appropriate patient safety topics for inclusion in undergraduate training of health professionals?
b. In your view how did the students respond to patient safety teaching included in the curriculum?
c. Can you suggest ways that this Curriculum Guide can be used to support the widespread implementation of explicit patient safety education globally?
d. How could the Curriculum Guide be modified in the future to best support teaching of patient safety to students in different environments?

Privacy and Confidentiality

Participation in the evaluation will be voluntary. All interviews will be conducted at times convenient to the participants, and every step will be taken to accommodate reasonable times to conduct the interview.

All data will be collected, stored and managed with strict confidentiality. Stringent safeguards will be taken to ensure confidentiality and anonymity for all interview/group discussion data across schools/universities. A secure WHO telephone line will be used to conduct all interviews. Interview data that is gathered from '*Implementation coordinators*' will be securely stored in password protected computers.

Written consent will be sought from all stakeholders prior to the start of interviews or discussion groups. Permission will also be sought to allow taking minutes from the interviews/group discussions. If a participant declines permission, his/her interview will not be recorded. Interviewees will be provided a Word file containing a summary of their interviews and offered the chance to comment and verify that the log is accurate.

All logs from the interviews will be de-identified. No interview data will be listed by name and no comparison will be made across schools/universities.

Interview Questions

1. Contents of the Curriculum Guide

<u>1.1.Topics and Approach</u>

Did the Curriculum Guide inform your organization about the key topics in patient safety?

Does the range of topics in the Curriculum Guide reflect the relative importance of safety issues in your country?

Are there any additional topics that should be addressed in the Curriculum Guide?

Are there any topics that should be deleted or reduced in importance?

Do you have any suggestions to improve the Patient Safety Topics in the Curriculum Guide?

<u>1.2 Support for Educators (Part A)</u>

Overall, how helpful is the Curriculum Guide for educators to develop capacity and skills to teach patient safety?

How effective is the Curriculum Guide in assisting educators to integrate patient safety learning into their curricula?

How culturally appropriate for your country are the contents of the Curriculum Guide? (methods, skills and knowledge)

How appropriate are the assessment strategies provided in the curriculum guide for the resources and teaching environment in your country?

How appropriate are the evaluation strategies in the Curriculum Guide for evaluating the patient safety courses

<u>1.3 Course Content (Part B)</u>

How helpful to you was the overall content of the Curriculum Guide topics in teaching patient safety to your students?

(Note which are being taught in that school:)

a) dentistry students
b) nursing students
c) midwifery students
d) pharmacy students

2. Experiences Implementing Curriculum Guide

Is patient safety already being taught in your school?

Which Curriculum Guide topics are already being taught at your school? Why were these topics chosen?

How were decisions made to use the Curriculum Guide for teaching patient safety and for choices of specific topics?

Who was involved in making these decisions and how were they involved?

Probe: institutional leadership, faculty, students, others
Probe: seminars, meetings, focus groups, surveys of faculty

What priority did the institution’s leadership give to this initiative in patient safety teaching?
What priority did the institution’s faculty give to the WHO initiative in patient safety teaching?

What was your experience in obtaining agreements and approvals to teach the topics as part of the educational curriculum?

How did you introduce the selected patient safety topics? Were they taught as separate course(s), or were they integrated into existing curricula?

What did you do, if anything, to integrate teachings of patient safety topics across different clinical disciplines?

How do the techniques you used for the Curriculum Guide topics compare to how you deliver your existing curricula?

What methods did you use to assess students' learning in patient safety?

What were your experiences as you started to teach the topics? Of what? Experiences is a big word-

Suggest

Can you identify any specific issues or challenges you faced when you began to teach the topics?

How did your experience of teaching the patient safety curriculum compare to what you had expected?

What were your greatest successes or benefits in introducing the topics?

What were your greatest challenges in introducing the topics?

What changes did you make during the training to address issues that arose during the implementation of the Curriculum Guide topics?

Based on your experience with teaching these topics, how might you change your approach to teaching them in the future?

How did your faculty respond to evaluation of the patient safety topics introduced?

3. Usability of the Curriculum

How readily could you integrate the contents of each topic with actual practices and issues involved in professionals' care delivery roles?

How easy was it to introduce the Curriculum Guide topics selected into the Faculty's existing curricula?

How easy or difficult to understand is the language used in the Curriculum Guide:

for educators? for students?

Are the Curriculum Guide topics presented in a user-friendly format?

4. Acceptance of the Curriculum Guide

What if any was the response of colleagues and other educators to the Curriculum Guide?
Were colleagues supportive of teaching the Patient Safety topics?

If some faculty members were critical of the Curriculum Guide, what were the reasons for their criticisms?

What did the faculty see as the strengths of the Curriculum Guide?

What did the faculty see as the weaknesses of the Curriculum Guide?

In your view what was the response of the students to the Curriculum Guide?

For students who were critical of the Curriculum Guide, what were the reasons for their criticisms?

What did the students report as strengths of the Curriculum Guide?

What did the students report as weaknesses of the Curriculum Guide?

5. Value of Training Provided

Did the Curriculum Guide contribute to the knowledge base students require during professional training?

Which topics in the Curriculum Guide advance patient safety knowledge and practice? Which topics in the Curriculum Guide were least valuable?

Is the Curriculum Guide a sound educational investment, considering the knowledge value it offers and the resources involved in teaching it?

6. Effects on the Institution's Patient Safety Capacity

Before using the Curriculum Guide, did your institution include patient safety topics in the curricula?

How much does the Curriculum Guide contribute to strengthening the institution's capacity to teach patient safety?
What additional capacity would the institution like or need in order to strengthen its patient safety capacity?

7. Other Potential Applications for the Curriculum

In what other ways would you like to use the Curriculum Guide at your institution?

Do you think organizations apart from universities or schools that train health care professionals might find the curriculum useful.? If so which types of organizations?

How useful would the Curriculum Guide topics be is taught through e-courses to students?

What advice would you give to other schools as they are consider using of the Curriculum Guide?

What implementation guidance would you give to these schools as they start to teach the Curriculum Guide for their students?

Protocol for Teaching Faculty Interviews

Evaluation of the WHO Patient Safety Curriculum Guide, Multi-professional Edition

The following questions will inform the evaluation:

a. Does the curriculum guide contain the necessary and sufficient information and topics to allow its effective use in undergraduate training of health professionals?
b. What is the impact upon student learning of the inclusion of patient safety teaching in the Curriculum Guide?
c. In what ways can this Curriculum Guide be used to support the widespread implementation of explicit patient safety education globally?
d. How could the Curriculum Guide be modified in the future to best support teaching of patient safety to students in different environments?

Privacy and Confidentiality

Participation in the evaluation will be voluntary. All interviews will be conducted at times convenient to the participants, and every step will be taken to accommodate reasonable times to conduct the interview.

All data will be collected, stored and managed with strict confidentiality. Stringent safeguards will be taken to ensure confidentiality and anonymity for all interview/group discussion data across schools/universities. A secure WHO telephone line will be used to conduct all interviews. Interview data that is gathered from '*Teaching faculty*' will be securely stored in password protected computers.

Written consent will be sought from all stakeholders prior to the start of interviews or discussion groups. Permission will also be sought to allow taking minutes from the interviews/group discussions. If a participant declines permission, his/her interview will not be recorded. Interviewees will be provided a Word file containing a summary of their interviews and offered the chance to comment and verify that the log is accurate.

All logs from the interviews will be de-identified. No interview data will be listed by name and no comparison will be made across schools/universities.
that would cause loss of reputation, embarrassment or compromise confidentiality.

Interview Questions

1. Contents of the Curriculum Guide

<u>1.1 Topics and Approach</u>

Did the Curriculum Guide inform your organization about the key topics in patient safety?

Does the range of topics in the Curriculum Guide reflect the relative importance of safety issues in your country?

What additional topics, if any, should be addressed in the Curriculum Guide?

What topics if any should be deleted or reduced in importance?

Do you have any suggestions to improve the Patient Safety Topics in the Curriculum Guide?

1.2 Support for Educators (Part A)

Overall, how helpful is the Curriculum Guide for educators to develop capacity and skills to teach patient safety?

How effective is the Curriculum Guide in assisting educators to integrate patient safety learning into their curricula?

How effective is the Curriculum Guide in informing educators on:

Importance of building supportive environments for teaching patient safety
How to design and implement patient safety curricula
The appropriate methods and tools for planning teachings
The appropriate methods and tools for assessing students' learning
The use of inter-disciplinary teaching in patient safety

How culturally appropriate for your country are the contents of the Curriculum Guide? (methods, skills and knowledge)

Are the assessment strategies outlined in the curriculum appropriate given the resources and teaching environment in your country?

Are the evaluation strategies in the Curriculum Guide appropriate for evaluating the patient safety courses?

What suggestions do you have to improve the Teacher's Guide?

1.3 Course Content (Part B)

How helpful was the overall content of the Curriculum Guide topics in teaching patient safety to your students?

(Note which are being taught in that school:)

a) dentistry students
b) nursing students
c) midwifery students
d) pharmacy students

Which of the following elements in teaching the patient safety topics were helpful?

Multi-professional teaching content
Learning objectives
Knowledge requirements
Performance requirements
Teaching slides
Patient safety resources

2. Experiences Implementing Curriculum Guide

Is patient safety already being taught in your school?

Which Curriculum Guide topics if any were already being taught at your school? Why did you select these topics?

How did you decide to use the Curriculum Guide for teaching patient safety and how did you select the specific topics?

Who was involved in making these decisions and how were they involved?
Probe: institutional leadership, faculty, students, others
Probe: seminars, meetings, focus groups, surveys of faculty

What priority did the institution's leadership give to this initiative in patient safety teaching?

What priority did the institution's faculty give to this initiative in patient safety teaching?

What was your experience in obtaining agreements and approvals to teach the topics as part of the educational curriculum?

How did you introduce the selected patient safety topics? Were they taught as separate course(s), or were they integrated into existing curricula?

What did you do, if anything, to integrate teachings of patient safety topics across different clinical disciplines?

What mix of teaching techniques did you use (balance of lecture, small group discussion, guest speakers, simulation, learning activities)?

How do the techniques you used for the Curriculum Guide topics compare to how you deliver your existing curricula?

Were local experiences, case studies, or literature incorporated in teaching patient safety topics?

What methods did you use to assess students' learning?

What were your experiences as you started to teach the topics?

How did those experiences compare to what you had expected?

What were your greatest successes in introducing the topics?

What were your greatest challenges in introducing the topics ?

What changes did you make during the training to address issues that arose during the implementation of the Curriculum Guide topics?

Based on your experience with teaching these topics, how might you change your approach to teaching them in the future?

How did students respond to their sessions on patient safety?

How did students respond to the teaching methods used?

What methods did you use to assess student's learning?

How did students respond to performance assessment methods?

How did faculty respond to evaluation of the patient safety topics introduced?

3. Usability of the Curriculum

How readily could you integrate the contents of each topic with actual practice?

How adaptable were the contents of the Curriculum Guide topics to local cultural needs and requirements?

Did you think the topics were evidence-based?

Do you think the content of the topics helped students understand how they could use what they learned in future professional practices/actions?

How easy was it to introduce the Curriculum Guide topics selected into your existing curricula?

How useful were each of the teaching resources provided in the guide:

- The slides for teaching the topics
- Literature on the patient safety topic
- Examples provided
- Case studies
- The tools

Which tools did you use? For those not used, why did you not use them?

How clear is the language used in the Curriculum Guide?

for educators? for students?

Are the Curriculum Guide topics presented in a user-friendly format?

4. Acceptance of the Curriculum Guide

What was the response of colleagues and other educators to the Curriculum Guide? How supportive were they of teaching the Patient Safety topics?

For faculty who were critical of the Curriculum Guide, what were the reasons for their criticisms?

What did the faculty see as the strengths of the Curriculum Guide?

What did the faculty see as the weaknesses of the Curriculum Guide?

What was the response of the students to the Curriculum Guide?

For students who were critical of the Curriculum Guide, what were the reasons for their criticisms?

What did the students see as the strengths of the Curriculum Guide?

What did the students see as the weaknesses of the Curriculum Guide?

5. Value of Training Provided

Did the Curriculum Guide contribute to the knowledge base students require during their professional training?

Have the patient safety courses had an impact on current student clinical performance in terms of practicing safe care?

Which topics in the Curriculum Guide advance patient safety knowledge and which topics if any in the Curriculum Guide topics are the least valuable?

Is the Curriculum Guide a sound investment considering the knowledge value it offers and the resources involved in teaching it?

6. Effects on Students' Knowledge and Motivation

Do you think students gained a greater understanding of the importance of patient safety as a result of the training they received?

Do you think students' knowledge of patient safety principles increased as a result of the training they received?

Do you think students' knowledge of patient safety practices increased as a result of the training they received?

Do you think students gained a greater understanding of how to apply patient safety practices in their professional roles?

Do you think students' gained a stronger motivation (intention) to put patient safety practices to work in their professional roles?

7. Effects on the Institution's Patient Safety Capacity

Before using the Curriculum Guide, did your institution include patient safety topics in the curricula? (curriculum)

How much does the Curriculum Guide contribute to strengthening the institution's capacity to teach patient safety?

What additional capacity would the institution like or need in order to strengthen its patient safety capacity further?

8. Opportunities to Improve the Curriculum Guide

What do you think are strongest parts of the Curriculum Guide?

What do you think are the weakest parts of the Curriculum Guide?

What changes would you suggest to improve the weak parts identified?

What other changes would you suggest to improve the usability of the Curriculum Guide content, teaching instructions, slides, or tools?

9. Other Potential Applications for the Curriculum

In what other ways do you think you could use the Curriculum Guide at your institution?

Do you think organizations other than universities or schools that train health care professionals might find the Curriculum Guide useful? If so which types of organizations?

How useful would the Curriculum Guide topics be is taught through e-courses to students?

What advice would you give to other schools as they consider using the Curriculum Guide?

What implementation guidance would you give to these schools as they start to teach the Curriculum Guide for their students?
