# Supplementary material for: Field Test of the World Health Organization Multi-Professional Patient Safety Curriculum Guide
Source: PLoS One. 2015 Sep 25;10(9):e0138510. doi: 10.1371/journal.pone.0138510 (PMC4583458; doi:10.1371/journal.pone.0138510)
Supplement: S2 Text — (PDF) [file pone.0138510.s003.pdf]

S2 Text. Questionnaire for Post-Teaching Student Survey

<u>Attitude Questions</u> on Understanding of Patient Safety

WHO Patient Safety Curriculum Guide: Multi-professional Edition

Dear Colleague,

The Multi-professional Patient Safety Curriculum Guide is published by WHO to support education and training in patient safety in health-care universities and schools around the world. This questionnaire is designed to evaluate health-care students' awareness and knowledge of patient safety issues in the healthcare system.

We anticipate it will take around 10-15 minutes to complete the questionnaire. Please respond to the questions honestly, based on your current level of knowledge. If you prefer not to respond to a particular question then just leave it blank. The questionnaire is anonymous and any information you provide will be reported at a group level, not individually.

You are being asked to complete this form as part of a study. This will not form part of your final assessment. Completing it is not compulsory.

Introductory questions

1. What is your current year of study? (Please circle as appropriate)

1st year 2nd year 3rd year 4th year5^{th} year 6th year

2. Has patient safety been taught in your university/school? (Please circle as appropriate)

Yes No

3. Have you previously taken a patient safety course? (Please circle as appropriate)

Yes No

Section 1 Error and Patient safety

For questions in Section 1, please circle the most appropriate number according to a five point scale where: 1= low level of knowledge, 3=moderate level of knowledge, and 5= high level of knowledge.

What is your level of knowledge regarding	Low		Moderate		High
1. Different types of human error in health care?	1	2	3	4	5
2. Factors contributing to human error?	1	2	3	4	5
3. Factors influencing patient safety?	1	2	3	4	5
4. Ways of speaking up about error?	1	2	3	4	5
5. What should happen if an error is made?	1	2	3	4	5
6. How to report an error?	1	2	3	4	5
7. The role of health-care organizations (e.g. hospitals, general practitioners) in error reporting?	1	2	3	4	5

For questions from Section 2 to 4, please circle the most appropriate number according to a five point scale where: 1 = strongly disagree, 2 = disagree, 3 = neutral (neither agree nor disagree), 4 = agree and 5 = strongly agree.

Section 2 Safety of the Healthcare System

Please circle the number that best describes your level of agreement for each statement.

	Strongly Disagree	Disagree	Neutral	Agree	Strongly Agree
1. Most health-care workers make errors.	1	2	3	4	5
2. In my country there is a safe system of healthcare for patients.	1	2	3	4	5
3. Medical error is very common.	1	2	3	4	5
4. It is very unusual for patients to be given the wrong drug.	1	2	3	4	5
5. Health-care staff receive training in patient safety.	1	2	3	4	5

Section 3 Personal Influence over Safety

Thinking about your own ability to influence patient safety, please circle the number that best describes your personal view for each statement.

	Strongly Disagree	Disagree	Neutral	Agree	Strongly Agree
1. Telling others about an error I made would be easy.	1	2	3	4	5
2. It is easier to find someone to blame rather than focus on the causes of error.	1	2	3	4	5
3. I am confident about speaking to someone who is showing a lack of concern for a patient's safety.	1	2	3	4	5
4. I know how to talk to people who have made an error.	1	2	3	4	5
5. I am always able to ensure that patient safety is not compromised.	1	2	3	4	5
6. I believe that filling in reporting forms will help to improve patient safety.	1	2	3	4	5
7. I am able to talk about my own errors.	1	2	3	4	5

Section 4 Personal Attitudes to Patient Safety

Thinking about your personal attitudes with regard to patient safety, please circle the number that best describes your own attitude for each statement.

	Strongly Disagree	Disagree	Neutral	Agree	Strongly Agree
1. By concentrating on the causes of incidents I can contribute to patient safety.	1	2	3	4	5
2. If I keep learning from my mistakes, I can prevent incidents.	1	2	3	4	5
3. Acknowledging and dealing with my errors will be an important part of my job.	1	2	3	4	5
4. It is important for me to learn how best to acknowledge and deal with my errors by the end of medical school.	1	2	3	4	5

Thank you for taking the time to complete the attitude questions

<u>Knowledge Questions</u> on Patient Safety Topics

WHO Patient Safety Curriculum Guide: Multi-professional Edition

You are being asked to complete this form as part of a study. This will not form part of your final assessment. Completing it is not compulsory.

Please circle the number that you think is the right answer for the question. It could be more than one right answer.

Topic 1: What is Patient Safety

A) What multiple factors can lead to the delivery of unsafe care?

1. latent factors: organizational process, high workloads, lack of leadership and etc.
2. research factors: measuring effectiveness of clinical protocol, randomized clinical trial
3. error-producing factors: busy ward, poor procedures and etc.
4. failures: unsupervised junior staff, communication difficulties and etc.
5. defences: missing medical history

B) A doctor fails to practice hand hygiene between patients because they feel they are too busy even if there is a alcohol handrub dispenser in the ward. This is an example of:

1. a patient safety system failure issue
2. a tolerated behavior in the ward
3. a routine violation
4. a lack of supervision issue

Topic 2: Why applying human factors is important for patient safety

A) What human factors predispose health-care workers to errors:

1. limited memory capacity
2. fatigue
3. stress
4. poor training
5. hunger
6. illness
7. culture/language factors
8. hazardous behaviors

B) How should we apply human factors thinking to health-care environment?

1. avoid relying memory
2. make things visible: use of reminders, notes, displays and etc.
3. routinely use checklists/standardize procedures
4. improve personal knowledge and skills
5. decrease reliance on vigilance: awareness of potential errors

C) Sandra had an episiotomy during the delivery process. She consulted her obstetrician due to vaginal smelling 10 days after the delivery. The obstetrician suspected a urinary tract infection and prescribed antibiotics. Sandra retuned to see her obstetrician a week later with the same symptoms. After checking on her delivery notes, the obstetrician found no errors and prescribed an additional course of antibiotics. As the symptoms persisted, Sandra went to see a different obstetrician who found a swab left behind during the packing of the episiotomy wound.

Please nominate human factors that may have contributed to leaving the swab behind:

1. reliance on memory when performing swab count
2. not following the standardized procedure in episiotomy
3. inexperienced health-care workers
4. wrong prescription by the obstetrician
5. fatigue or stress experienced by health-care workers
6. inadequate checking by the second nurse and the obstetrician

Topic 3: Understanding systems and the effect of complexity on patient care

A) What are the elements of a safe health-care delivery system?

1. well trained health-care workers
2. acknowledging the possibility of 'failure'
3. seeking out 'risks' and containing them before they cause harm
4. personal accountability
5. initiating and maintaining a safety culture
6. involving patient in the care

B) Please outline elements of a 'system-thinking' approach to address adverse events:

1. patient and the health-care workers involved
2. technology and tools used

3. related organization policy
4. team communications
5. blame and shame health-care workers
6. organizational factors: workflow, time pressure, workload of health-care workers

C) Which model below best describes the system approach in addressing adverse events in health-care environments.

1. health belief model
2. swiss cheese model
3. stages of change model
4. perfectibility model

Topic 4: Being an effective team player

A) The TeamSTEPPS programme identifies a number of different, but interrelated team types that support and deliver health care. Teams formed for emergency or specific events belong to which type of the team below:

1. core team
2. coordinating team
3. contingency team
4. ancillary service
5. support serve

B) There are four stages in team development. Open communication between team members is established in stage:

1. Forming
2. Storing
3. Norming
4. Performing

C) As a student, you have been invited to observe a knee replacement in an elderly female. The day before the operation, you talked to the patient and remembered being told that trouble with her left knee had made it impossible for her to walk and that she was looking forward to having it fixed. In the operating theatre, you hear the surgeon say to his assistant that they are going to be operating on her right knee.

What should you, as a student, do next?

1. do nothing because you may have confused this patient with another patient.
2. locate and review the medical records to confirm the side for the knee replacement.
3. say nothing because hospitals never make mistakes and the student probably misheard.
4. tell the surgeon that you thought that the patient was having her left knee replaced.
5. keep silent because the surgeon is likely to know what he is doing.

Topic 5: How we understand and learn from errors to prevent harm

A) Wrong diagnosis leads to an inappropriate treatment plan. What type of error is it?

1. skill-based slips and lapses
2. violations
3. rule-based mistakes
4. knowledge-based mistakes

B) A patient was being treated for non-Hodgkin's lymphoma. A Syringe containing vincristine for another patient has been accidently delivered to patient's bedside. The physician administered vincristine via a spinal route, believing it was a different medication. The error was not recognized and the patient died three days later. What are the major factor(s) that caused the error in this case?

1. inexperience
2. poor procedures
3. inadequate checking and patient identification
4. inadequate information

C) What are the best way to learn from errors?

1. criticize the health-care workers involved in the incident
2. establish incident reporting and monitoring system
3. never report near misses as they do not incur any harm
4. limit reporting on adverse events to prevent health-care workers being blamed or published
5. root cause analysis could be used to review sentinel incidents

Topic 6: Understanding and managing clinical risk

A) Baby Edward, a preterm infant with respiratory distress requiring ventilation, was prescribed intravenous gentamicin for a serious infection. The intensive-care nurse and pediatrician were extremely busy. The drug was administered 90 minutes later than prescribed. Fortunately, Edward survived and recovered.

1) Should this incident (near miss) be reported?

1. Yes 2. No

2) If yes, which method should you apply to report the incident?

1. discussed as a standing item at the weekly staff meetings
2. discuss the fact about the incident and follow-up action required is done with the team
3. discussion focusing on attributing blame
4. identify system-related issues involved in the incident

B) How should patient complaints handled?

1. discuss complaints openly with patients and their families
2. health-care workers to who the complaint is directed is blamed and reprimanded
3. necessity of a factual statement related to health-care workers involved with the complaint
4. policy for managing complaints is in place

Topic 7: Using quality-improvement methods to improve care

A) Measurement is an essential component of quality improvement. What are the main types of measures:

1. outcome measures
2. demographic measures
3. process measures
4. balancing measures

B) Clinical practice improvement (CPI) is used to improve the quality and safety of health care. The success of a CPI project depends on the team covering five phases. In which phase will the team gather as much information about the problem as possible and make the decision on how to measure the improvement?

1. project phase
2. diagnostic phase
3. intervention phase
4. impact and implementation phase
5. sustaining and improvement phase

Topic 8: Engaging with patients and caregivers

A) Which are major phases for gaining an informed consent from patients?

1. disclosure of information by health-care workers
2. competence of health-care workers
3. free and voluntary choice made by patients
4. accountability of health-care workers and the organization
5. time allowed for patient to absorb the information disclosed and consult with family/carers
6. comprehension of the information by the patient

B) Which are the key principles of open disclosure?

1. openness and timeliness of communication
2. support of staff
3. punishment and compensation
4. acknowledgement of the incident
5. confidentiality
6. admission of responsibility

Topic 9: Infection prevention and control

A) List the five key moments to practice hand hygiene.

1. before entering the ward
2. before touching a patient
3. before a clean/aseptic procedure
4. after bodily fluid exposure risk
5. after touching a patient
6. after touching patient surroundings

B) What are the main types of health-care associated infection?

1. urinary tract infections usually associated with catheters
2. surgical infections
3. food related infections
4. blood stream infections associated with the use of an intravascular device
5. pneumonia associated with ventilators

C) What are the main transmission routes for health care-associated infections?

1. transmission through direct and indirect contact
2. transmission through food
3. droplet transmission
4. airborne transmission

D) How do you dispose sharps safely?

1. discard each needle into a sharps' container at the point of use
2. do not overload the bin for 'sharps' if it is full
3. recap, bend or break needles after use
4. do not leave a sharps' bin in the reach of children

Topic 10: Patient safety and invasive procedures

A) List the three main element of the WHO Patient Safety Surgical checklist.

1. before enter operating room
2. before induction of anesthesia
3. before skin incision
4. before patient leaves operation room

B) A male patient was admitted for removal of his right kidney. The operating list stated 'left' by mistake. The patient was not woken from sleep to check the correct side on the preoperative ward round. The side was not checked with the notes or the consent form. The consultant surgeon put the correctly labeled X-rays on the view box back to front. The senior registrar surgeon began to remove the left kidney. A medical student observing the operation suggested to the surgeon that he was removing the wrong kidney, but was ignored. The mistake was not discovered and led to the death of the patient.

Please identify the missing opportunities for checking the site of surgery.

1. patient should NOT be woken up to confirm on the operating side
2. patients' medical documents and consent form should be double checked before operating
3. the consultant surgeon should check X-ray carefully
4. the registrar surgeon should trust the consultant surgeon without a double check on the operating side
5. student's suggestion should be considered

Topic 11: Improving medication safety

A) There are four steps in using medication. The four steps are: prescribing, dispensing, administering and monitoring.

1) Identify factors to prescription errors:

1. considering individual patient factors, such as allergies, pregnancy, co-morbidities, other medications
2. wrong patient, wrong dose, wrong time, wrong drug, wrong route
3. look-a-like and sound-a-like medications
4. Inadequate communication (written, verbal)
5. documentation issues, such as illegible, incomplete, ambiguous
6. mathematical error when calculating dosage

2) Identify factors to monitoring errors?

1. lack of monitoring for side-effects
2. drug not ceased if not working, or course completed
3. wrong patient, wrong dose, wrong time, wrong drug, wrong route
4. drug levels not measured, or not followed up
5. communication failures

B) Which methods below contribute to safer medication use?

1. use trade names for medicine as they are more commonly known by patients
2. encourage patients to be actively involved
3. depends on your memory when prescribing medication
4. tailor prescribing for individual patients
5. communicate clearly
6. never use high-risk medications

C) An old man saw a community doctor for treatment of new onset stable angina. After taking a full past history and medication history, the doctor discovers the patient has been healthy and only takes medication for headaches every day. The doctor assumes it is an analgesic as the patient cannot recall the name of the headache medication. But the medication is actually a beta-blocker. The doctor prescribed aspirin and another beta-blocker for the angina. The new medication leads to bradycardia and postural hypotension of the patient.

What should you do to prevent this error from occurring?

1. make sure to collect complete and accurate medication history

2. never make assumption

2. no need to inform patient about his/her medications

3. trust patient about his/her medication history even without seeing the medication list

5. warn the patient about potential side-effects and what to do if side-effects occur

Thank you for taking the time to complete the knowledge questions

<u>Feedback Questions</u> on the Patient Safety Topic Taught in this Course

WHO Patient Safety Curriculum Guide: Multi-professional Edition

You are being asked to complete this form as part of a study. This will not form part of your final assessment. Completing it is not compulsory.

Introductory questions

Which patient safety topics have you been taught? (Please mark all that apply)

□ What is patient safety?

□ Why applying human factors is important for patient safety

□ Understanding systems and the effect of complexity on patient care

□ Being an effective team player

□ Learning from errors to prevent harm

□ Understanding and managing clinical risk

□ Using quality-improvement methods to improve care

□ Engaging with patients and caregivers

□ Infection prevention and control

□ Patient safety and invasive procedures

□ Improving medication safety

For questions in Section 1 and 2, please circle the most appropriate number according to a five point scale where:

1 = strongly disagree, 2 = disagree, 3 = neutral (neither agree nor disagree),

4 = agree and 5 = strongly agree.

Section 1 Perception about the Patient Safety Topics

Please respond to the following questions regarding your perceptions about the patient safety topic taught in this course.

	**Strongly Disagree**	**Disagree**	**Neutral**	**Agree**	**Strongly Agree**
1. The aims of this patient safety topic were clear to me.	1	2	3	4	5
2. It was important to incorporate this patient safety training in the educational curricula.	1	2	3	4	5
3. I have improved my knowledge/skills as a result of this topic.	1	2	3	4	5
4. I acquired new knowledge/skills that will be of value during my career.	1	2	3	4	5
5. I will be able to readily apply the knowledge taught in the Curriculum Guide to actions during my professional work.	1	2	3	4	5
6. My understanding of the importance of patient safety increased as a result of the patient safety training.	1	2	3	4	5
7. My knowledge of patient safety principles and practices increased as a result of the patient safety training.	1	2	3	4	5
8. This training increased my motivation to put patient safety practices to work in my professional roles.	1	2	3	4	5

Section 2 Effectiveness of the Patient Safety Topics

Please respond to the following questions regarding how effectively the patient safety topic was taught in this course.

	Strongly Disagree	Disagree	Neutral	Agree	Strongly Agree
1. I found the style of teaching in this topic facilitated my learning.	1	2	3	4	5
2. The Instructors facilitated my understanding.	1	2	3	4	5
3. The presentation of the topic was culturally appropriate.	1	2	3	4	5
4. The teaching aids *(e.g. audiovisual presentations or written materials)* added to the session (please only answer this question if aids were used).	1	2	3	4	5
5. Completion of the assignment(s) facilitated my understanding (please only answer this question if you were given assignments).	1	2	3	4	5
6. The time devoted to the topic was sufficient.	1	2	3	4	5
7. The methods used to assess performance on the topic taught were effective.	1	2	3	4	5
8. This stage in the overall Curriculum Guide is an appropriate time for this particular topic.	1	2	3	4	5

Section 3 Comments on Patient Safety Teaching

Complete the following questions with free text. We value any comments you have about the topic(s). These will be used to further develop teaching in the future.

1. Please indicate any areas that you considered particularly worthwhile in the taught topic(s).

2. Please indicate any areas that you would like changed or improved in the taught topic(s).

3. Any other comments about the taught topics would be most welcome.

Thank you for taking the time to complete this questionnaire
