# Supplementary material for: Field Test of the World Health Organization Multi-Professional Patient Safety Curriculum Guide
Source: PLoS One. 2015 Sep 25;10(9):e0138510. doi: 10.1371/journal.pone.0138510 (PMC4583458; doi:10.1371/journal.pone.0138510)
Supplement: S3 Text — (PDF) [file pone.0138510.s004.pdf]

S3 Text. Criteria for Selecting Participating Pilot Sites

Participation as a pilot site to the evaluation study is voluntary. Following WHO policies and the external peer review process, criteria for qualifying each pilot site have been developed, which was approved by the WHO Ethics Review Committee (ERC) in October 2011. The criteria were shared with the patient safety focal points at each of six WHO regional offices to recruit qualified university/school to participate in the evaluation study. The selection criteria are listed as below:

1. The Faculty or School is a Dentistry, Midwifery, Nursing and Pharmacy Faculty, or School within a University.
2. University, Faculty, or School is recognized by a national approving or regulatory body (e.g. Ministry of Education or Health) or certification from the relevant credentialing national body.
3. University, Faculty, or School will provide in writing to WHO evidence of local approvals including Ethics Review approvals for testing the Multi-professional WHO Patient Safety Curriculum Guide in a timely manner and will be prepared to start the class within 6 months after receiving the finalized Curriculum Guide.
4. University, Faculty, or School agrees to review all the topics of the Curriculum Guide and to implement and teach at least 4 out of the 11 topics for the pilot study.
5. University, Faculty, or School is willing to participate in the evaluation and to appoint a contact person to serve as liaison with the WHO evaluators. It agrees to perform the tasks identified in the Evaluation Information Note (Available at WHO website: http://www.who.int/patientsafety/education/curriculum/Information_Note-3_evaluation_2012-Jan.pdf?ua=1 accessed July 29, 2015).
6. University, Faculty or School has demonstrated an interest in teaching patient safety topics.
