# Supplementary material for: Field Test of the World Health Organization Multi-Professional Patient Safety Curriculum Guide
Source: PLoS One. 2015 Sep 25;10(9):e0138510. doi: 10.1371/journal.pone.0138510 (PMC4583458; doi:10.1371/journal.pone.0138510)
Supplement: S4 Text — (PDF) [file pone.0138510.s005.pdf]

S4 Text. Informed Consent Forms

Separate 'Informed Consent' forms for institutional executives, implementation coordinators, teaching faculty and students are provided here.

1. 'Informed Consent' form for institutional executives;
2. 'Informed Consent' form for implementation coordinators and teaching faculty;
3. 'Informed Consent' form for students

[YOUR INSTITUTIONAL LETTER HEAD]
INSTITUTIONAL EXECUTIVES

Informed Consent Form for institutional executives who are invited to participate in the global evaluation study on the WHO Patient Safety Curriculum Guide: Multi-professional Edition.

Name of Principal Investigator: Dr Agnès Leotsakos and Dr Hao Zheng
Name of Organization: World Health Organization
Name of Sponsor: None
Name of Project and Version: WHO Patient Safety Curriculum Guide: Multi-professional Edition Pilot Study V 19

This Informed Consent Form has two parts:

- **Information Sheet (to share information about the study with you)**
- **Certificate of Consent (for signatures if you choose to participate)**

You will be given a copy of the full Informed Consent Form and fact sheet

Part I: Information Sheet

Introduction

I, ………………………………, would like to invite you to help evaluate the Patient Safety Curriculum Guide: Multi-professional Edition developed by the World Health Organization. This form briefly outlines the global evaluation study of the Curriculum Guide. It also highlights the support we request from you in terms of supporting its evaluation. This evaluation is to assess the value and effectiveness of the Curriculum Guide as a resource for teaching patient safety to undergraduate health-care students of dentistry, midwifery, nursing and pharmacy disciplines.

The Curriculum Guide is composed of two parts: Part A: Teacher's Guide and Part B: 11 patient safety topics. It is designed to be easily integrated into existing health-care education curricula using a flexible approach to meet individual needs. The 11 topics of the Curriculum Guide are stand alone modules and are presented in the attached fact sheet (Annex 1). The topics address various areas in patient safety ranging from what is patient safety and the importance of human factors in patient safety, to infection control and medication safety. More details on the aims of the Curriculum Guide including the development process are provided in Annex 1. Additional information on the Curriculum Guide can be obtained from the WHO website by clicking on this link: http://www.who.int/patientsafety/education/curriculum/en/index.html.

I would like to invite you to be part of this evaluation study in your role as Dean/Head/Director of this school. Please do not hesitate to ask me via email if you need additional information or if you have any questions.

Purpose of the research

The purpose of this research is to evaluate the Patient Safety Curriculum Guide: Multi-professional Edition. The Curriculum Guide is designed to be an educational resource for health-care schools/universities for teaching patient safety at the undergraduate level. The global evaluation study is designed to answer the following questions:

a. Does the Curriculum Guide contain the necessary and sufficient topics and information to

allow its effective use in undergraduate training of health professionals?

b. What is the impact upon student learning of the inclusion of patient safety teaching in the Curriculum Guide?
c. In what ways can this Curriculum Guide be used to support the widespread implementation of explicit patient safety education globally?
d. How could the Curriculum Guide be modified in the future to best support teaching of patient safety to students in different environments?

Type of Research Intervention
This evaluation study will involve your participation in one individual phone interview that will take approximately 30 to 45 minutes. In addition to the interview time, we anticipate that perhaps an extra 30 minutes would be needed to review the information about this study and discuss it with university faculty. Discussion points will be shared before the interview.

Participant Selection
Your experience as a leader in health care education will greatly contribute in this global evaluation .

Voluntary Participation
It is up to you to decide whether or not to support this evaluation study. You may change your mind later and stop participating even if you agreed earlier.

Procedures
We are inviting you to take part in this evaluation study as your experience as a leader in health care education will greatly contribute to this work. If you agree to participate, you will be interviewed by one of the Principal Investigators (PIs) or a WHO Curriculum Guide Consultant. During the interview, questions will be asked relating to your executive insight on the topics of the WHO Patient Safety Curriculum Guide, their appropriateness and usability, as well as the acceptance of the Curriculum Guide and its impact on the institution's capacity to teach patient safety. Minutes will be taken from the interview, and interviewees will be provided a Word file containing a summary of the interview and offered the chance to comment and verify that the interpretation is accurate. Telephone calls will be scheduled in advance and the PIs will be sensitive that call times are reasonable for participation from different time zones.

Duration
The study takes place over 18 months in total. You will be interviewed when your school has completed implementation of the patient safety topics. This interview will last between 30 to 45 minutes.

Risks
The risks associated with participation in this research are very low. Principally these risks could potentially pertain to maintaining confidentiality, anonymity and use of data that might directly or indirectly affect the institution's reputation. The potential of these risks has been appropriately dealt with and explained in the relevant sections below. There are no other major risks associated with your participation.

Benefits
There will be no direct benefit to you, but your insight and experience is likely to help improve the content and delivery of the Multi-professional edition of the WHO Patient Safety Curriculum Guide.

Reimbursements
No incentives or reimbursement will be provided by participating in this global study.

Confidentiality

The information that we collect on the interview will be kept private. Only PIs and WHO Curriculum Guide team members will have access to your identity and interview data. Papers and notes of interviews will be kept under lock and key. Only authorized individuals on the project (PIs, Consultant and WHO Curriculum Guide team members) will have access to these electronic files. Access logs will be maintained electronically.

Sharing the Results
No data at the individual or institutional level will be shared with anyone outside the WHO Curriculum Guide team. Reports and publications that will arise from this study will never identify any individual or institution. The PIs and WHO Curriculum Guide team will make all efforts to ensure that interview data that might allow for indirect linking to the institution will not be shared publicly. We will forward you the summary of your interview before it is analysed and get the opportunity to modify and correct any discrepancies. The pilot sites will be duly acknowledged for their contribution in all reports and publications arising from this study.

Right to Refuse or Withdraw
You have the right to refuse to participate in this study and may stop participating in the discussion/interview at any time that you wish. There is an opportunity at the end of the interview/discussion to review your remarks, and you can ask to modify or remove portions of those, if you do not agree with the notes or if the interviewer did not understand correctly.

Who to Contact
If you have any questions, you can ask me or our local contact (to be provided) now or later.

PI Contact information: Dr Agnès Leotsakos
WHO Patient Safety Programme
Avenue Appia 20
1211 Geneva 27
Switzerland
work: +41 22 791 2567
email: leotsakosa@who.int

Local Contact: to be provided

This proposal has been reviewed by the Ethics Review Committee of the World Health Organization (WHO), which is supporting the study.

Part II: Certificate of Consent

I have been invited to participate in this global evaluation study to assess the effectiveness of the WHO Patient Safety Curriculum Guide: Multi-professional Edition in health-care education.

I have read the foregoing information. I have had the opportunity to ask questions about it and any questions I have been asked have been answered to my satisfaction. I consent voluntarily to be a participant in this study.

Print Name of Participant__________________
Signature of Participant ___________________
Date ___________________________
Day/month/year

[YOUR INSTITUTIONAL LETTER HEAD]
IMPLEMENTATION COORDINATORS AND TEACHING FACULTY

Informed Consent Form for implementation coordinators and teaching faculty who are invited to participate in the global evaluation study on the WHO Patient Safety Curriculum Guide: Multi-professional Edition.

Name of Principal Investigator: Dr Agnès Leotsakos and Dr Hao Zheng
Name of Organization: World Health Organization
Name of Sponsor: None
Name of Project and Version: WHO Patient Safety Curriculum Guide: Multi-professional Edition Pilot Study V 19

This Informed Consent Form has two parts:

- **Information Sheet (to share information about the study with you)**
- **Certificate of Consent (for signatures if you choose to participate)**

You will be given a copy of the full Informed Consent Form and fact sheet

Part I: Information Sheet

Introduction

I, ………………………………. , would like to invite you to help evaluate the Patient Safety Curriculum Guide: Multi-professional Edition developed by the World Health Organization. This form briefly outlines the global evaluation study of the Curriculum Guide. It also highlights the support we request from you in terms of supporting its evaluation. This evaluation is to assess the value and effectiveness of the Curriculum Guide as a resource for teaching patient safety to undergraduate health-care students of dentistry, midwifery, nursing and pharmacy disciplines.

The Curriculum Guide is composed of two parts: Part A: Teacher's Guide and Part B: 11 patient safety topics. It is designed to be easily integrated into existing health-care education curricula using a flexible approach to meet individual needs. The 11 topics of the Curriculum Guide are stand alone modules and are presented in the attached fact sheet (Annex 1).The topics address various areas in patient safety ranging from what is patient safety and the importance of human factors in patient safety, to infection control and medication safety. More details on the aims of the Curriculum Guide including the development process are provided in Annex 1. Additional information on the Curriculum Guide can be obtained from the WHO website by clicking on this link: http://www.who.int/patientsafety/education/curriculum/en/index.html.

I would like to invite you to be part of this evaluation study in your role as faculty educator or professor at this school and support this work by introducing and teaching selected patient safety topics from the WHO Patient Safety Curriculum Guide: Multi-professional Edition. Please do not hesitate to ask me via email if you need additional information or if you have any questions.

Purpose of the research
The purpose of this study is to evaluate the Patient Safety Curriculum Guide: Multi-professional Edition. The Curriculum Guide is designed to be an educational resource for health-care schools/universities for teaching patient safety at the undergraduate level. The global evaluation study is designed to answer the following questions:

a. Does the Curriculum Guide contain the necessary and sufficient topics and information to allow its effective use in undergraduate training of health professionals?
b. What is the impact upon student learning of the inclusion of patient safety teaching in the Curriculum Guide?
c. In what ways can this Curriculum Guide be used to support the widespread implementation of explicit patient safety education globally?
d. How could the Curriculum Guide be modified in the future to best support teaching of patient safety to students in different environments?

Type of Research Intervention
This evaluation study involves introducing and teaching students selected patient safety topics from the WHO Patient Safety Curriculum Guide. After the courses have been completed, all 'Teaching faculty' will be invited to participate in a telephone group interview. The group interview will last about 1-1.5 hours. The purpose of this group interview is to get feedback on the value, effectiveness and impact of the Curriculum Guide and what needs to be improved. Faculty invited to participate in the group interview will be required to review the content of the selected topics taught for suitability and local adaptation. The review time will vary based on the number for topics selected to be implemented by the school and the assigned faculty. We estimate the review time to be between 2- 4 hours per topic. This includes review of the Teacher's Guide (Part A) that introduces patient safety concepts to faculty and provides guidance on the planning and design of the courses. It also includes review of the selected patient safety topics (Part B) including all teaching materials and resources supplied. In addition, you will be invited to participate in WHO's online Community of Practice to share your experiences with others as you implement the Curriculum Guide on an ongoing basis. This is an internet-based discussion forum that will allow the WHO Curriculum Guide team to gather feedback on individual knowledge, experiences, lessons-learned from the implementation of the Curriculum Guide. If you are the 'Implementation coordinator', you will be invited to participate in two additional individual telephone interviews with the Principal Investigators (PIs) and WHO Curriculum Guide team members to share your experiences on the process of implementing the Curriculum Guide. The first interview will be conducted at the start of the courses and the second upon their completion. Each interview will last about 1 hour. Discussion points will be shared before the interview, and their review will require an additional 1 hour. You will also be invited to participate in WHO's online Community of Practice to share your experiences with others as you coordinate the implementation of the Curriculum Guide on an ongoing basis. The 'Implementation coordinator' will facilitate enrolment and participation by relevant faculty members in the WHO Community of Practice that will be established by WHO as part of the pilot testing.

Participant Selection
Your experience as a professor and health-care educator will greatly contribute in this global evaluation.

Voluntary Participation
It is up to you to decide whether or not to support this evaluation study. You may change your mind later and stop participating even if you agreed earlier.

Procedures
We are inviting you to take part in this evaluation study as your experience as an health-care educator will greatly contribute to this work. If you agree to participate, you will be asked to join a group interview and the online WHO Community of Practice. There will be an additional individual interview for 'Implementation coordinators'. Minutes will be taken from the interview/group discussion, and interviewees will be provided a Word file containing a summary of the interview/group discussion and offered the chance to comment to verify that the interpretation is accurate. Telephone calls will be scheduled in advance and the project PIs will be sensitive that call times are reasonable for participation from different time zones.

A few examples of questions that will be posed are given below.

Example for Teaching faculty group interview
- How helpful is the Curriculum Guide for educators to develop capacity and skills to teach patient safety?
- How effective is the Curriculum Guide in assisting educators to integrate patient safety learning into their curricula?
- How culturally appropriate for your country are the contents (topics) of the Curriculum Guide ?

Example for phone interviews for implementation coordinators
- How were decisions made to use the Curriculum Guide for teaching patient safety and for the choices of the specific topics?
- Who was involved in making these decisions and how were they involved?
- What was your experience in obtaining agreements and approvals to teach the topics as part of the existing curricula?
- How well does the Curriculum Guide cover the important patient safety topics?

WHO Community of Practice
An internet-based community of practice will be established. Faculty can post their experiences and questions to each other on an ongoing basis. As discussion threads grow these will be categorized under topics to make it easier to collaborate. The instructions on how to enroll and participate in this internet-based discussion forum will be shared with all participants via email. The WHO Curriculum Guide team will assist and provide additional training to faculty if needed.

Duration
The study takes place over 18 months in total. 'Implementation coordinators' will be invited to participate in an individual phone interview in the first week that the Curriculum Guide topics are being taught, and the other one in the second week after the courses are done. Each interview will last for about one hour. The group interview for 'Teaching faculty' will be held after the last pilot site has completed teaching the Curriculum Guide topics. This will last between 1-1.5 hours.

Risks
The risks associated with participation in this research are very low. Principally these risks could potentially pertain to maintaining confidentiality, anonymity and use of data that might directly or indirectly affect the institution's reputation. The potential of these risks has been appropriately dealt with and explained in the relevant sections below. There are no other major risks associated with your participation.

Benefits
There will be no direct benefit to you, but your insight and educator's experience will be instrumental in helping improve the content and delivery of the Multi-professional edition of the WHO Patient Safety Curriculum Guide.

Reimbursements
No incentives or reimbursement will be provided by participating in this global study.

Confidentiality
The information collected on the interview will be kept private. Only PIs and WHO Curriculum Guide team members will have access to your identity and interview data. Papers and notes of interviews/group discussions will be kept under lock and key. Only authorized individuals on the project (PIs, Consultant and WHO Curriculum Guide team members) will have access to these electronic files. Access logs will be maintained electronically.

The only place where you will be identified by other participating sites is the online WHO Community of Practice. The Community of Practice is a private discussion and collaboration space shared amongst professors and educators of all participating pilot sites to engage in frank and open discussion about their implementation experience and opinions in real time. Access to this site is by invitation and only faculty members involved in this study will be able to view this information.

Collected data from group interviews and online discussion will be de-identified and any reporting will be based on pooled data to maintain confidentiality. The PIs and WHO Curriculum Guide team will take due precautions to ensure individual level interview data is not shared.

We will ask all participants in the group interview and online discussion not to talk to people outside the group about what was said in the group or posted within online forum. We will, in other words, ask each of you to keep what was said or posted in the group confidential. However, we cannot stop or prevent participants who were in the group from sharing things that should be confidential.

Sharing the Results
No data at the individual or institutional level will be shared with anyone outside the WHO Curriculum Guide team. Reports and publications that will arise from this study will never identify any individual or institution. The PIs and WHO Curriculum Guide team will make all efforts to ensure that interview data that might allow for indirect linking to the institution will not be shared publicly. We will forward the summary of your interview before it is analysed and get the opportunity to modify and correct any discrepancies. The pilot sites will be duly acknowledged for their contribution in all reports and publications arising from this study.

Right to Refuse or Withdraw
You have the right to refuse to participate in this study and may stop participating in the discussion/interview at any time that you wish without any affect whatsoever on the school. There is an opportunity at the end of the interview/discussion to review your remarks, and you can ask to modify or remove portions of those, if you do not agree with the notes or if the interviewer did not understand correctly.

Who to Contact
If you have any questions, you can ask me or our local contact (to be provided) now or later.

PI Contact information: Dr Agnès Leotsakos
WHO Patient Safety Programme
Avenue Appia 20
1211 Geneva 27
Switzerland
work: +41 22 791 2567
email: leotsakosa@who.int

Local Contact: to be provided

This proposal has been reviewed by the Ethics Review Committee of the World Health Organization (WHO), which is supporting the study.

Part II: Certificate of Consent

I have been invited to participate in this global evaluation study to assess the effectiveness of the WHO Patient Safety Curriculum Guide: Multi-professional Edition in health-care education.

I have read the foregoing information. I have had the opportunity to ask questions about it and any questions I have been asked have been answered to my satisfaction. I consent voluntarily to be a participant in this study.

Print Name of Participant__________________
Signature of Participant ___________________
Date ___________________________
Day/month/year

[YOUR INSTITUTIONAL LETTER HEAD]
STUDENTS

Informed Consent Form for university students who take the courses using the WHO Patient Safety Curriculum Guide: Multi-professional Edition.

Name of Principal Investigator: Dr Agnès Leotsakos and Dr Hao Zheng
Name of Organization: World Health Organization
Name of Sponsor: None
Name of Project and Version: WHO **Patient Safety Curriculum Guide: Multi-professional Edition Pilot Study V 19**

This Informed Consent Form has two parts:

- **Information Sheet (to share information about the study with you)**
- **Certificate of Consent (for signatures if you choose to participate)**

You will be given a copy of the full Informed Consent Form and Fact sheet

Part I: Information Sheet

Introduction
I, ………………………………. , would like to invite you to help evaluate the Patient Safety Curriculum Guide: Multi-professional Edition developed by the World Health Organization. This form briefly outlines the global evaluation study of the Curriculum Guide. It also highlights the support we request from you in terms of supporting its evaluation. This evaluation is to assess the value and effectiveness of the Curriculum Guide as a resource for teaching patient safety to undergraduate health-care students of dentistry, midwifery, nursing and pharmacy disciplines.

The Curriculum Guide is composed of two parts: Part A: Teacher's Guide and Part B: 11 patient safety topics. It is designed to be easily integrated into existing health-care education curricula using a flexible approach to meet individual needs. The 11 topics of the Curriculum Guide are stand alone modules and are presented in the attached fact sheet (Annex 1).The topics address various areas in patient safety ranging from what is patient safety and the importance of human factors in patient safety, to infection control and medication safety. More details on the aims of the Curriculum Guide including the development process are provided in Annex 1. Additional information on the Curriculum Guide can be obtained from the WHO website by clicking on this link: http://www.who.int/patientsafety/education/curriculum/en/index.html.

I would like to invite you to be part of this evaluation study as student at this school. Your school has agreed participate in this study and will be teaching you courses on patient safety topics from the WHO Patient Safety Curriculum Guide: Multi-professional Edition. You do not have to decide today whether or not you will participate in this study. Before you decide, you can talk to anyone you feel comfortable with about this study. Please do not hesitate to ask me via email if you need additional information or if you have any questions.

Purpose of the research
The purpose of this study is to evaluate the Patient Safety Curriculum Guide: Multi-professional Edition. The Curriculum Guide is designed to be an educational resource for health-care schools/universities for teaching patient safety at the undergraduate level. The global evaluation study is designed to answer the

following questions:

a. Does the Curriculum Guide contain the necessary and sufficient topics and information to allow its effective use in undergraduate training of health professionals?
b. What is the impact upon student learning of the inclusion of patient safety teaching in the Curriculum Guide?
c. In what ways can this Curriculum Guide be used to support the widespread implementation of explicit patient safety education globally?
d. How could the Curriculum Guide be modified in the future to best support teaching of patient safety to students in different environments?

Type of Research Intervention
This evaluation study will involve your participation in two paper-based surveys that will take about 10-15 minutes for each to be completed. The first survey will be given to you when you start the course and the second survey will be given to you at the end of the course.

Participant Selection
Your experience as a health-care student will greatly contribute in this global evaluation.

Voluntary Participation
It is up to you to decide whether or not to support this evaluation study. The choice that you make will have no bearing on your study or on any study-related evaluations or reports. You may change your mind later and stop participating even if you agreed earlier.

Procedures
We are inviting you to take part in this evaluation study as your experience as a health-care student will greatly contribute to this work. If you agree to participate, you will be asked to respond to two paper-based surveys. The questions that will be asked pertain to topics and content of courses , their relevance and usefulness.

Examples of the questions in surveys
- Did you gain a greater understanding of the importance of patient safety as a result of the patient safety courses you received?
- How did the training topics from the WHO Patient Safety Curriculum Guide affect your perceptions regarding the relative importance of specific patient safety issues?

Duration
The study takes place over the duration of time that you will be taught this course. Should you decide to participate, you will be requested to complete two surveys. One on the first week of class and the second one during the last week of the class. Each survey will take approximately 10 – 15 minutes to complete.

Risks
The risks associated with participation in this research are very low. Principally these risks could potentially pertain to confidentiality, anonymity, sharing of results as described in the sections below. Your participation in this study will not affect your academic scores in any way. There are no other known risks associated with your participation. You would be committing a total of 20-30 minutes of your school time.

Benefit
There will be no direct benefit to you, but your participation is likely to help us find out how effective the Curriculum Guide is for health-care education and where additional changes are still needed.
Reimbursements

No incentives or reimbursement will be provided by participating in this global study.

Confidentiality

You will not be asked to provide your name on the survey. We will request you to generate a secret code that you will use when answering the survey questions both times. We request that you not share your secret code with anyone. The information collected from surveys will be kept private. Any information about you will have just the secret code on it instead of your name. All answered surveys will be kept under lock and key.

Sharing the Results

No data from surveys will be shared with anybody outside the WHO Curriculum Guide team, and nothing will be attributed to you by name. The knowledge that we get from this study will be shared with your university/school before it is made widely available to the public. Neither faculty, nor PIs nor the WHO Curriculum Guide team will be able to identify your pre- and post- results.

Right to Refuse or Withdraw

You have the right to refuse to participate in this study and may stop participating in the survey at any time that you wish without any effect on your student or any other role you may have.

Who to Contact

If you have any questions, you can ask me or our local contact (to be provided) now or later.

PI Contact information: Dr Agnès Leotsakos
WHO Patient Safety Programme
Avenue Appia 20
1211 Geneva 27
Switzerland
work: +41 22 791 2567
email: leotsakosa@who.int

Local Contact: to be provided

This proposal has been reviewed by the Ethics Review Committee of the World Health Organization (WHO), which is supporting the study.

Part II: Certificate of Consent

I have been invited to participate in this global evaluation study to assess the effectiveness of the WHO Patient Safety Curriculum Guide: Multi-professional Edition in health-care education.

I have read the foregoing information. I have had the opportunity to ask questions about it and any questions I have been asked have been answered to my satisfaction. I consent voluntarily to be a participant in this study.

Print Name of Participant__________________
Signature of Participant ___________________
Date ____________________________
Day/month/year
